# Emaciated enigma: Decline in body conditions of common dolphins in the Celtic Seas ecoregion

**DOI:** 10.1002/ece3.70325

**Published:** 2024-09-30

**Authors:** Sofia Albrecht, Cóilín Minto, Emer Rogan, Rob Deaville, Jim O'Donovan, Mags Daly, Stephanie Levesque, Simon Berrow, Andrew Brownlow, Nicholas J. Davison, Orla Slattery, Luca Mirimin, Sinéad Murphy

**Affiliations:** ^1^ Marine and Freshwater Research Centre, Department of Natural Resources and the Environment, School of Science and Computing Atlantic Technological University Galway Ireland; ^2^ Department of Zoology, Ecology and Plant Science University College Cork Ireland; ^3^ Institute of Zoology Zoological Society of London London UK; ^4^ Department of Agriculture, Food and the Marine Regional Veterinary Laboratory Cork Ireland; ^5^ Irish Whale and Dolphin Group Kilrush Ireland; ^6^ Scottish Marine Animal Stranding Scheme, School of Biodiversity, One Health and Veterinary Medicine College of Medical, Veterinary and Life Sciences University of Glasgow Glasgow UK

**Keywords:** blubber thickness, body condition, common dolphin, health, marine mammal, nutrition

## Abstract

Monitoring the nutritional health of cetaceans has become increasingly important in a changing environment, where multiple stressors impact animals. Particularly for those species that require consumption of energy‐dense prey, such as the common dolphin. Thus far, no uniform measure for monitoring body condition has been recommended across cetaceans, and species‐specific measures may need to be developed if employed as a population condition indicator under Descriptor 1 of the Marine Strategy Framework Directive. Here, nine morphometric body condition indices were applied to long‐term common dolphin stranding data sets originating from Ireland and the UK. We report a recent decline in the nutritional health of common dolphins in the Celtic Seas ecoregion comparing animals from 2017 to 2019 to animals from 1990 to 2006, with an increase in cases of animals dying due to starvation. Using ordinal regression trees, ventral blubber thickness (VBT) was identified as the most important index to predict nutritional status, defined at necropsy, followed by the scaled mass index (SMI). Using generalised linear models, both the VBT and SMI indices differentiated individuals that died from chronic and acute causes of death (i.e., bycatch), where animals in chronic conditions had significantly thinner VBT and lower SMI. Both significant temporal and seasonal patterns in VBT were identified, with poorer body conditions observed during the autumn and better body conditions observed during the spring, as well as an overall decline detected in VBT during the study period. While VBT was positively correlated with total body length, SMI showed the opposite trend. The VBT index is recommended for monitoring nutritional health within the species when total body length and season are considered. Further research is needed to understand the underlying causes for the observed decline, including shifts in prey availability and/or quality, to inform targeted conservation management strategies.

## Graphical abstract

The nutritional health of stranded common dolphins in the Celtic Seas ecoregion has recently declined, as evidenced by a decrease in nutritional status and an increase in starvation‐related deaths. Ventral blubber thickness was identified as the most reliable predictor of nutritional status when comparing various morphometric indices using ordinal regression tree analysis. We recommend using ventral blubber thickness while considering body length and seasonal variations to monitor the health of these dolphins and to inform conservation strategies.

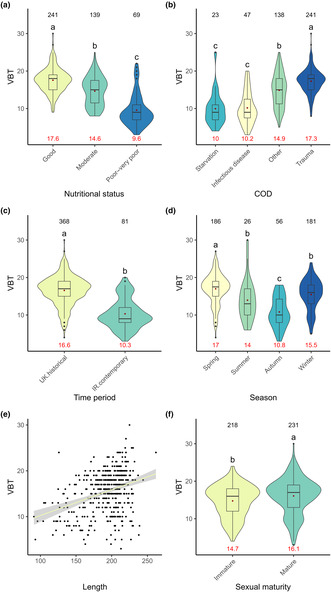

## 1 INTRODUCTION

Through the past decades, anthropogenic impacts have been increasingly affecting the Atlantic Ocean, with increasing potential being predicted for the future (Hansen et al., 2016; Lambert et al., 2014; Simmonds & Isaac, 2007). Overfishing and environmental change have altered marine ecosystems in Europe (Froese et al., 2018; Heide‐Jørgensen et al., 2011; Perry et al., 2005; Víkingsson et al., 2014), with the resulting impacts on prey availability and nutritional health of wild animals becoming a focus of conservation research (Kershaw et al., 2017; MacLeod et al., 2007; Murphy, 2015). Nutritional stress in marine mammals has been defined as the negative physiological and/or behavioural state resulting from reduced prey quality and/or quantity (Trites & Donnelly, 2003). Evaluating the potential impacts of nutritional stress at the regional and population level has been contentious, given the difficulty in disentangling the effects of one or more stressors on these species (Derous et al., 2020). A decrease in fitness has however been associated with changing prey communities/fishery‐linked resource availability in a range of marine mammal species, including baleen whales such as the southern right whale (*Eubalaena australis*) (Vermeulen et al., 2023) and North Atlantic fin whale (*Balaenoptera physalus*) (Williams et al., 2013), and toothed whales such as the killer whale (*Orcinus orca*) (Esteban et al., 2016; Ford et al., 2010; Jordaan et al., 2023), and pinnipeds, including the Steller sea lion (*Eumetopias jubatus*) (Trites, 2021).

The short‐beaked common dolphin (*Delphinus delphis*) is one of the most abundant top predators in the North Atlantic, playing a key functional role in marine ecosystems (Murphy et al., 2013, 2019; Pierce et al., 2022). Increasing numbers of stranded common dolphins have been reported along the Irish coastline in recent years (Levesque & Berrow, 2024; McGovern et al., 2018), many of whom were in an emaciated condition (Levesque et al., 2021). Previously, female common dolphins in the North‐east (NE) Atlantic population were reported to be in relatively good nutritional health, with no significant difference in nutritional status observed between animals that died during the 1990s (63% good, 29% moderate, and 8% poor nutritional condition) compared to those that died during the early 2000s (55% good, 37% moderate, and 8%, poor nutritional condition) (Murphy et al., 2009).

Common dolphins and other small cetaceans have high energetic demands of foraging and their life history (Spitz et al., 2010, 2012). They have been found to feed selectively on a few main species that vary with season and region (Brophy et al., 2009; Fariñas‐Bermejo et al., 2023; Murphy et al., 2013; Spitz et al., 2012). This has been attributed to an individuals' preference for feeding on energy‐dense prey, for example, prey species from the families Myctophidae, Carangidae, Argentinidae and Clupeidae, even if they are not the most abundant species (Brophy et al., 2009; Murphy et al., 2013; Spitz et al., 2010, 2012). For example, during the summertime, common dolphins in the Bay of Biscay consumed a higher proportion of lancet fish (*Notoscopelus kroeyeri*) which was in relatively low abundance compared to other myctophids, but of a higher energy density (Spitz et al., 2010). Such foraging strategies may be a combination of the costs of widespread feeding as well as the high energetic demands of reproduction, including pregnancy and lactation in females, and the seasonal development of enlarged testicular tissue in males for investment in post‐mating competition, that is, sperm competition (Murphy et al., 2005, 2013). Thus, the common dolphin may likely experience nutritional stress if a decline occurs in the availability of energy‐dense prey.

Assessments of body condition aimed at quantifying available energy reserves can be used to evaluate evidence of nutritional stress (Castrillon & Bengtson Nash, 2020; Krebs & Singleton, 1993; Pitt et al., 2006). Reserves result from energy uptake following total energetic expenditure (Peig & Green, 2010), the latter including foraging and life history‐related activities, such as reproduction (Aguilar & Borrell, 1990; Castrillon & Bengtson Nash, 2020; Jeanniard‐Du‐dot et al., 2017). In cetaceans, those reserves are stored in the form of adipose tissue in the blubber organ (Derous et al., 2020). However, the organ of blubber exhibits more roles than just being an energy reserve, such as structural roles in thermal insulation, hydrodynamics, buoyancy as well as mechanical energy storage during locomotion (Derous et al., 2020; Koopman, 1998; Noren & Wells, 2009). With overall body condition influencing individual growth rate, reproductive success and survival, body condition indices have thus been used as surrogates of fitness or fitness‐related traits (e.g., MacLeod et al., 2007; Murphy, 2015; Negri et al., 2014). Several body morphometric measures have been evaluated for assessing body condition in cetaceans (e.g., blubber thickness, body mass, girth, and body length) (Castrillon & Bengtson Nash, 2020; Gómez‐Campos et al., 2011; Hart et al., 2013; Kershaw et al., 2017; Murphy, 2015). However, no standardised established measure, applicable across different species and age groups, has been recommended so far, largely due to the differing functional requirements of blubber by different species. Measures have been recommended at a population and/or species level, depending on sample sources (i.e., live/dead) (Castrillon & Bengtson Nash, 2020). The use of morphometric data to assess overall body condition has been employed previously for common dolphins in the North‐west Atlantic (Joblon et al., 2014). In New England, live and dead stranded individuals were used to develop a visual scoring system for employment in photometric studies (Joblon et al., 2014). Other work undertaken by Sharp et al. (2014) on the same population reported that the body length‐to‐body‐girth ratio showed a significant difference between common dolphins that survived incidences of live strandings, compared to those that did not. Evaluation of a marine mammal blubber thickness metric has been undertaken by the Helsinki Commission (HELCOM) for consideration as a core indicator for biodiversity assessment (HELCOM, 2018). For harbour porpoises (*Phocoena phocoena*), grey (*Halichoerus grypus*) and harbour seals (*Phoca vitulina*) in the Baltic Sea, it was recommended that blubber thickness should only be used as a metric for assessing population condition when caveats including season, year of sampling, sex and age group are considered (Siebert et al., 2022).

During necropsies, the body conditions of common dolphins are classified into nutritional status categories (after Deaville & Jepson, 2011; Jepson et al., 2005), offering poor resolution. The development of a body condition indicator with relatively minor analytical costs (i.e., not based on direct measures of animal composition, see Wilder et al. (2016)) would enable the detailed evaluation of long‐term demographic changes in the NE Atlantic common dolphin population. Such a population condition indicator could be considered by international management bodies such as the OSPAR Commission, as a marine mammal biodiversity common indicator for reporting on the environmental status of the NE Atlantic, and also by EU Member States for reporting under descriptor 1 (biological diversity) of the Marine Strategy Framework Directive (MSFD), which evaluates the environmental status of European marine waters (2008/56/EC) (Murphy et al., 2019). To assist in this task, we applied all relevant cetacean body condition indices to valuable long‐term common dolphin datasets in the Celtic Seas ecoregion. These datasets originated from large‐scale collaborative stranding and observer bycatch programmes that spanned over three decades. Through the use of morphometric measurements, we aimed to identify the body condition indicators that best predicted nutritional status assessed at necropsy. Moreover, we assessed temporal trends in nutritional condition, and factors that may predict such, including sex, total body length, season, sexual maturity status, month found, and cause of death using generalised linear models. Work that will aid our understanding of current conservation issues and assist in targeting conservation efforts more effectively.

## 2 METHODS

### 2.1 Data collection

**FIGURE 1:**
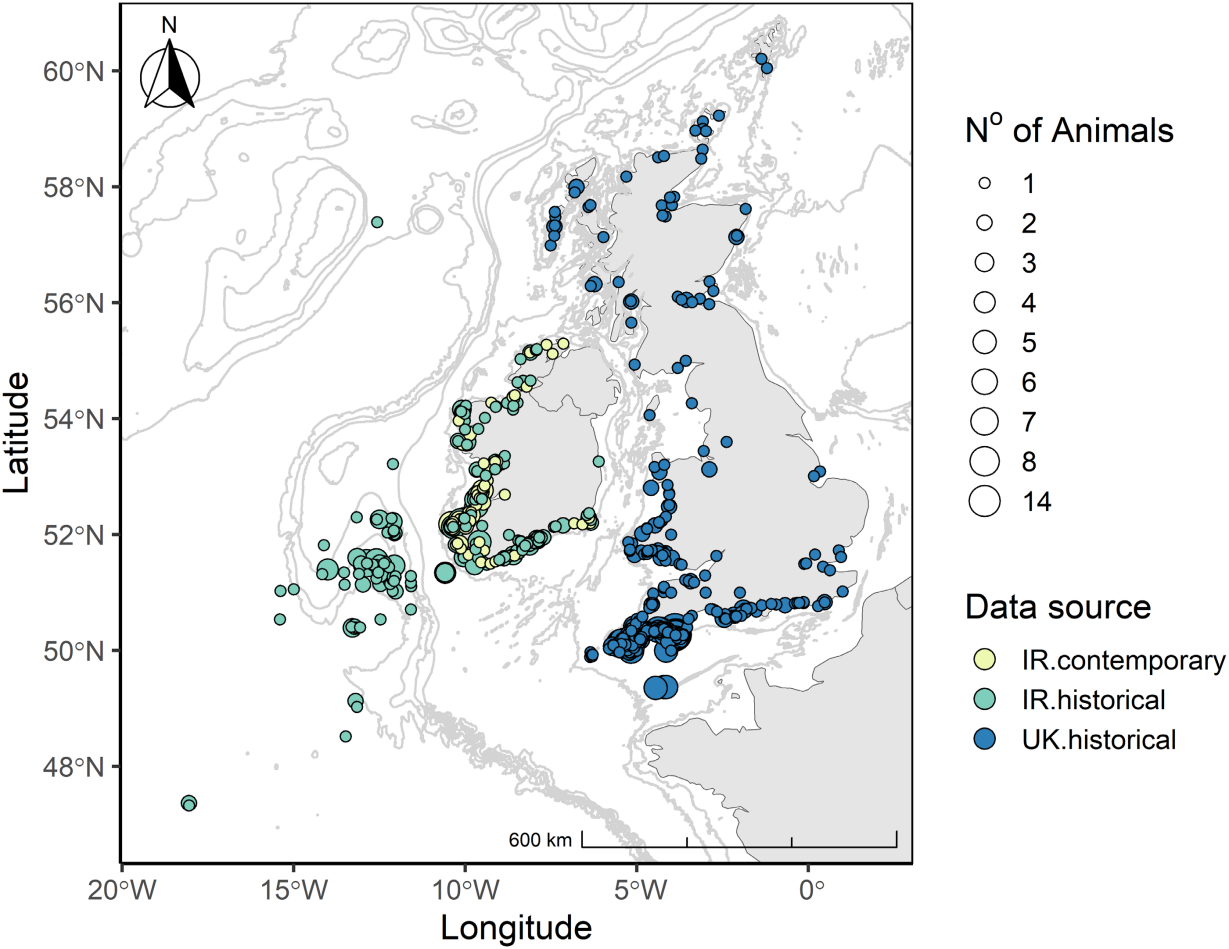
Stranding locations of common dolphins; Irish contemporary sampling period (2017–2019) indicated by yellow circles, Irish historical sampling period (1990–2004) indicated by green circles and the UK historical sampling period (1990–2006) indicated by blue circles. The number of animals stranded in one location is displayed through differences in the radius of the indicator points.

Common dolphins that were stranded and bycaught around the coasts of Ireland, Scotland, England and Wales were collected for postmortem examination (Figure 1). The Irish dataset comprised two sampling periods; 1990 and 2004 (*n* = 318) and 2017 and 2019 (*n* = 84). Additional data were sourced from stranded and bycaught common dolphins necropsied in the UK (*n* = 525, 1990–2006, Figure 1), data previously published in Murphy et al. (2009).

### 2.2 Sampling

All post‐mortems followed standardised procedures, recording sex, total body length (cm), ventral blubber thickness (mm, VBT) taken in front of the dorsal fin along the ventral aspect, and body girth (cm) in front of the dorsal fin (Bennett et al., 2000; Deaville & Jepson, 2011; Kuiken & García Hartmann, 1991; Levesque et al., 2021; Sabin et al., 2005; SACVSD, 2000). Animals included in the current study were classified into four decomposition status categories, including extremely fresh (*n* = 350), slight decomposition (*n* = 107), moderate decomposition (*n* = 290), and advanced decomposition (*n* = 58) (Deaville & Jepson, 2011; Kuiken & García Hartmann, 1991; Levesque et al., 2021). Nutritional status was assessed based on external examination of carcasses, and individuals were classified as; good: the aspect of the upper flanks on either side of the dorsal fin was rounded; moderate: the aspect of the upper flanks on either side of the dorsal fin is sloping; and poor to very poor: the aspect of the upper flanks on either side of the dorsal fin is hollow (Deaville & Jepson, 2011; Jepson et al., 2005). Causes of death were determined using specific diagnostic criteria, including gross, bacteriology, virology and histopathology assessments (Deaville & Jepson, 2011; Levesque et al., 2021), and individuals were grouped into the following four categories for the statistical analysis: trauma (bycatch, boat/ship strike, bottlenose dolphin attacks, and dystocia), infectious disease, starvation and others (e.g., live stranding, neoplasia and not established) (adapted from Murphy et al., 2015). Sexual maturity status was assessed via gonadal tissue analysis (*n* = 566, after Murphy et al., 2005, 2009). Where gonadal tissues were not assessed, individuals were classified as sexually immature or sexually mature based on estimates of the average body length and age at sexual maturity for the population (*n* = 264). For females, individuals were classified as sexually mature when equal to or larger than 188.8 cm in total body length, or equal to or older than 8.2 years in age (Murphy et al., 2009). Males were classified as sexually mature when equal to or larger than 206.5 cm, or equal to or older than 11.6 years (De Block, 2021). Age was determined based on dental growth layer analysis (Murphy et al., 2009; Murphy & Rogan, 2006).

### 2.3 Statistical analysis

#### 2.3.1 Changes in nutritional health over time

A primary objective of this study was to assess the nutritional health of common dolphins that stranded along Irish coastlines in recent years. We used an ordinal logistic regression to test for significant differences in the ordinal distribution of nutritional status across datasets. Therefore, the ordinal dependent variable was nutritional status and the explanatory variable was the data origin of the three previously described sampling periods (Irish contemporary, Irish historical and UK historical). The model was fit using “polr” in the MASS package in R (Venables & Ripley, 2002), and the Irish contemporary time period was set as the reference level.

#### 2.3.2 Selection of body condition indices for predicting nutritional status

Upon review of published body condition indices commonly employed for cetaceans (Table 1, Appendix S1), nine were selected for consideration within the current study. Their proficiency in predicting nutritional status as the response variable was determined via classification and regression tree analysis (CART) for ordinal responses using the package rpartScore (Galimberti et al., 2012). CART was selected as the analysis is deemed flexible and robust, can handle a broad range of response variable types, and deal with non‐linear relationships, complex interactions and missing values (De'Ath & Fabricius, 2000). All indices were included in the model, and the regression tree algorithm determined and prioritised their significance to identify optimal predictors. In this study, the dataset was randomly separated into an 80% training dataset (*n* = 743), which was used to build the model, and a 20% testing dataset (*n* = 184) used to evaluate the model's predictive performance. After the classification tree was estimated using the training dataset, it was pruned according to the lowest cross‐validation error with a standard deviation of the complexity parameter based on total misclassification cost, which ensures the most likely tree representing the data set (Galimberti et al., 2012). The model was validated by applying it to the testing data set and subsequently cross tabulating the observed and predicted nutritional status into a confusion matrix. Model residual diagnostic plots, variable importance and partial dependence profiles, were extracted from the selected tree via the package “moDel Agnostic Language for Exploration and eXplanation, DALEX” (Biecek, 2018). In detail, the variable importance rankings were extracted following the root mean square error (RMSE) loss after 100 permutations (Biecek, 2018; Breiman, 2001). Therefore, the decrease in the model's performance was measured when resampling values of the variable 100 times (Biecek, 2018; Breiman, 2001). To reduce the complexity of the tree generated through the training dataset the optimal tree was pruned. The position of pruning was defined through the lowest number of splits that contained the greatest complexity parameter. The average cross‐validated error of the chosen complexity parameter was within the standard error of the lowest cross‐validated error (De'Ath & Fabricius, 2000). Subsequently, the confusion matrix as well as variable importance plots and partial dependence profiles by the DALEX package were repeated using the pruned tree. Finally, suitable indices were chosen based on feature importance and partial dependence profiles of both trees.

**TABLE 1:** Body condition indices with their calculations and where those are taken from.

Index	Formula	Reference
Ventral blubber thickness (VBT)	Ventral blubber thickness	Derous et al. (2020), Ijsseldijk et al. (2021), Joblon et al. (2014), Kershaw et al. (2017), Koopman et al. (2002), Murphy (2015), Siebert et al. (2022)
Mass to body length (M/L)	$\frac{\text{Mass}}{\text{Length}}$	Karns et al. (2019), Kershaw et al. (2017), Williams et al. (2020, 2021)
Ventral blubber thickness to body length (VBT/L)	$\frac{\text{Ventral blubber thickness}\;}{\text{Length}}$	Kershaw et al. (2017)
Girth to body length (G/L)	$\frac{\text{Girth}\;}{\text{Length}}$	Castrillon and Bengtson Nash (2020), Heide‐Jørgensen et al. (2011), Kershaw et al. (2017)
d/r ratio (d/r)	$\frac{\text{Blubber thickness}}{\text{Girth}}$	Kershaw et al. (2017), Murphy (2015)
Body mass index (BMI)	$\mathrm{BMI}=\frac{\text{Mass}\;}{{\text{Length}}^{2}}$	Hart et al. (2013), Karns et al. (2019), Kershaw et al. (2017)
Residual index (Residual M/L)	The residuals from an OLS regression of Mass against Body length, after log transformation	Kershaw et al. (2017)
LMD‐index (LMD)	$\sqrt{\frac{\text{Body length}}{\text{Body weight}}}\times \text{Blubber tickness}\times 100$	Heide‐Jørgensen et al. (2011), Murphy (2015)
Scaled mass index (SMI)	$\hat{\mathrm{Mi}}=\mathrm{Mi}\times {\frac{L0}{\mathrm{Li}}}^{\text{bsma}}$ where Mi is the mass of an individual, Li is its body length, L0 is an arbitrary fixed body length and bSMA is the slope coefficient estimated from an SMA regression (Peig & Green, 2009)	Kershaw et al. (2017), Larrat and Lair (2021)

#### 2.3.3 Variables influencing body condition

After selecting VBT and scaled mass index (SMI), the best two body condition indices reflecting nutritional status, a Gaussian generalised linear model (GLM) was applied separately to each index, to determine if certain individuals within the population were more susceptible to nutritional stress. Explanatory variables included: cause of death, sex, sexual maturity status, total body length, nutritional status, country, month, season, quarter of the year, time period, and date reported. Seasons were defined as winter (November to January), spring (February to April), summer (May to July) and autumn (August to October). The quarter of the year was included as previous work undertaken on the species in Spain and Portugal reported that their diet and stranding rates varied by quartile (Marçalo et al., 2018; Santos et al., 2013). Nutritional status was included to confirm its correlation with the body condition index through a separate model. Only animals where all data were available were included within the GLM, which resulted in the exclusion of the Irish historical time period (due to a high number of missing data points for variables cause of death and nutritional status) as well as individual dolphin data within the other datasets. Model selection involved computing all possible models including all factor combinations, and the best models were identified by the lowest Akaike Information Criterion (AICc). Models within two units of the lowest AICc were considered and the final model was selected using the lowest AICc via the function dredge of the MuMin package (Barton, 2023). An additional model was built using the factors of the lowest AIC model including the two‐dimensional factor interactions. Adherence to model assumptions, such as the distribution of residuals, was assessed using the DHARMa package (Hartig, 2022). The multicollinearity of variables was checked using variance inflation factors populated through the “car” package (Fox & Weisberg, 2019). Thus, no variables had to be omitted due to multicollinearity.

Significant variables were identified using Chi‐square tests across the variances of the GLM model. Significant differences between factor levels were identified using Tukey HSD pairwise comparisons within the “agricolae” package (de Mendiburu & de Yaseen, 2020).

#### 2.3.4 Assessment of SMI applicability: Correlation with total body length

The SMI assumes that there is no correlation between the index and total body length (Larrat & Lair, 2021; Peig & Green, 2009). We tested this assumption using a Pearson's product–moment correlation test. The test analysed the relationship of the residuals of the standard major axis regression performed when calculating the index and the log total body length of the animals (Peig & Green, 2009).

Body mass and girth measures of pregnant females were excluded from all analyses. All statistical analysis and plots were performed using the free software package and interface RStudio (R version 4.2.2 (2022‐10‐31) – © 2022 The R Foundation for Statistical Computing). A significance level of <0.05 was employed.

## 3 RESULTS

### 3.1 Nutritional health of common dolphin in the Celtic Seas

**FIGURE 2:**
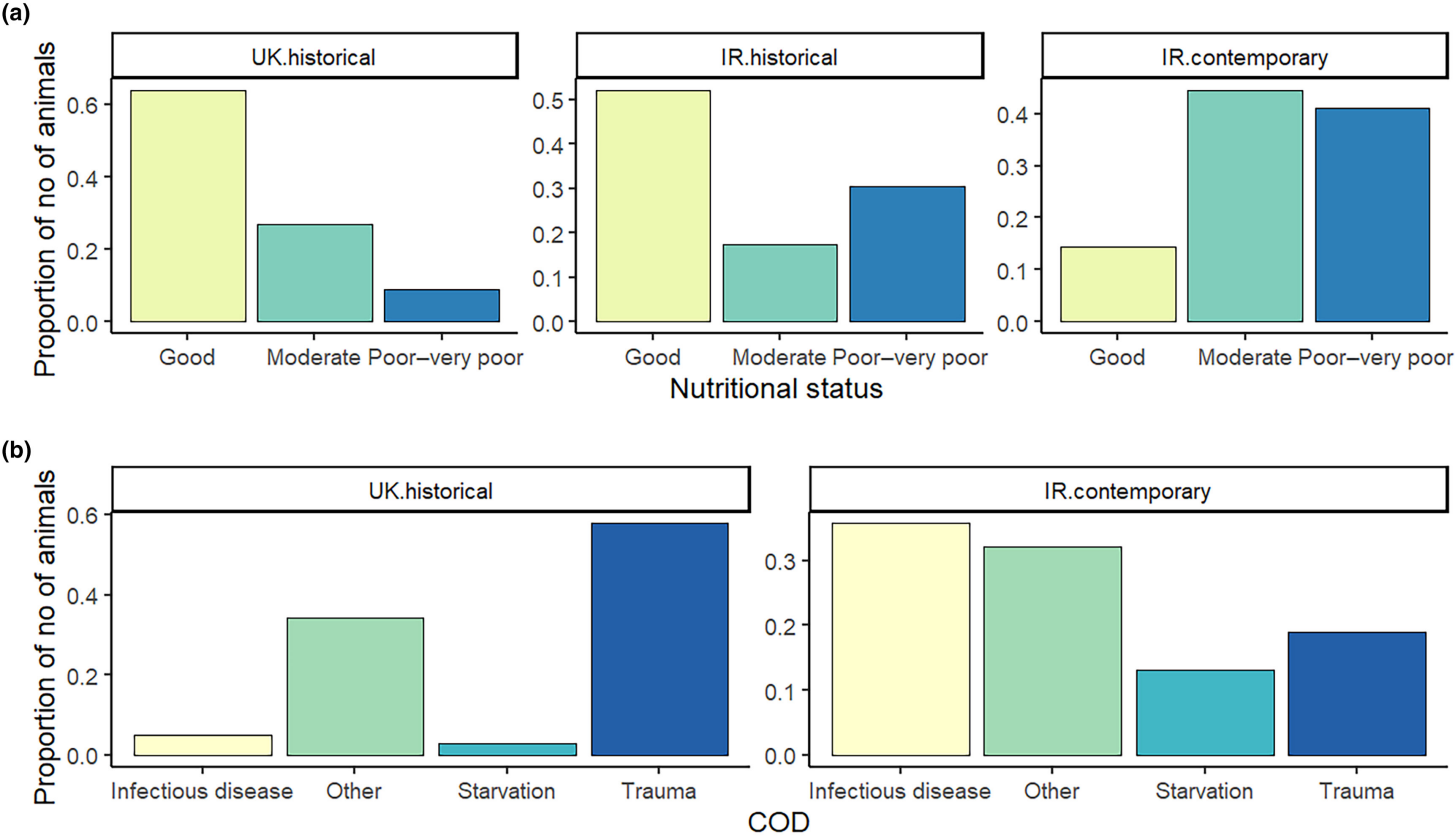
Relative frequency of (a) nutritional status categories for each time period, UK historical time period (*n* = 392), Irish historical (*n* = 23) and Irish contemporary (*n* = 83) and (b) COD categories for the UK historical (*n* = 467) and the Irish contemporary (*n* = 84).

A decline in the overall nutritional status of individuals was detected between time periods (UK 1990–2006, IR 1990–2004, and IR 2017–2019) employed within the current study (Figure 2). The relative proportion of individuals classified with good, moderate and poor‐to‐very poor nutritional status varied significantly between the Irish contemporary (the reference data set within the logistic regression), the Irish historical (*z* = −2.64, *p* = .01), and the UK historical (*z* = −8.66, *p* < .001) time periods (Table 2). There were significantly fewer animals with relatively good nutritional status in the Irish contemporary time period than in both the Irish and UK historical time periods (Figure 2a). Additionally, the proportion of stranded individuals dying due to starvation increased between the historical (UK data: 0.03, 14 out of 467 individuals) and the contemporary sampling periods (Irish data: 0.13, 11 out of 84 individuals, Figure 2b).

**TABLE 2:** Estimates for the logistic regression testing the differences for the nutritional status parameter between time periods, with the Irish contemporary time period as the reference level.

Time period	Estimate	Std. error	*z* value	Pr(>|*z*|)	Exp. coef, odd ratios	Lower CI	Upper CI
IR historical	−1.25	0.47	−2.64	0.01	0.29	0.11	0.72
UK historical	−2.08	0.24	−8.66	<0.001	0.13	0.08	0.20

*Note*: The IR historical and UK historical data are compared to the IR contemporary data and those model outputs are displayed. Model Call: Polr(formula = Nutritional status ~ Time period).

### 3.2 Selection of body condition indices for predicting nutritional status

**FIGURE 3:**
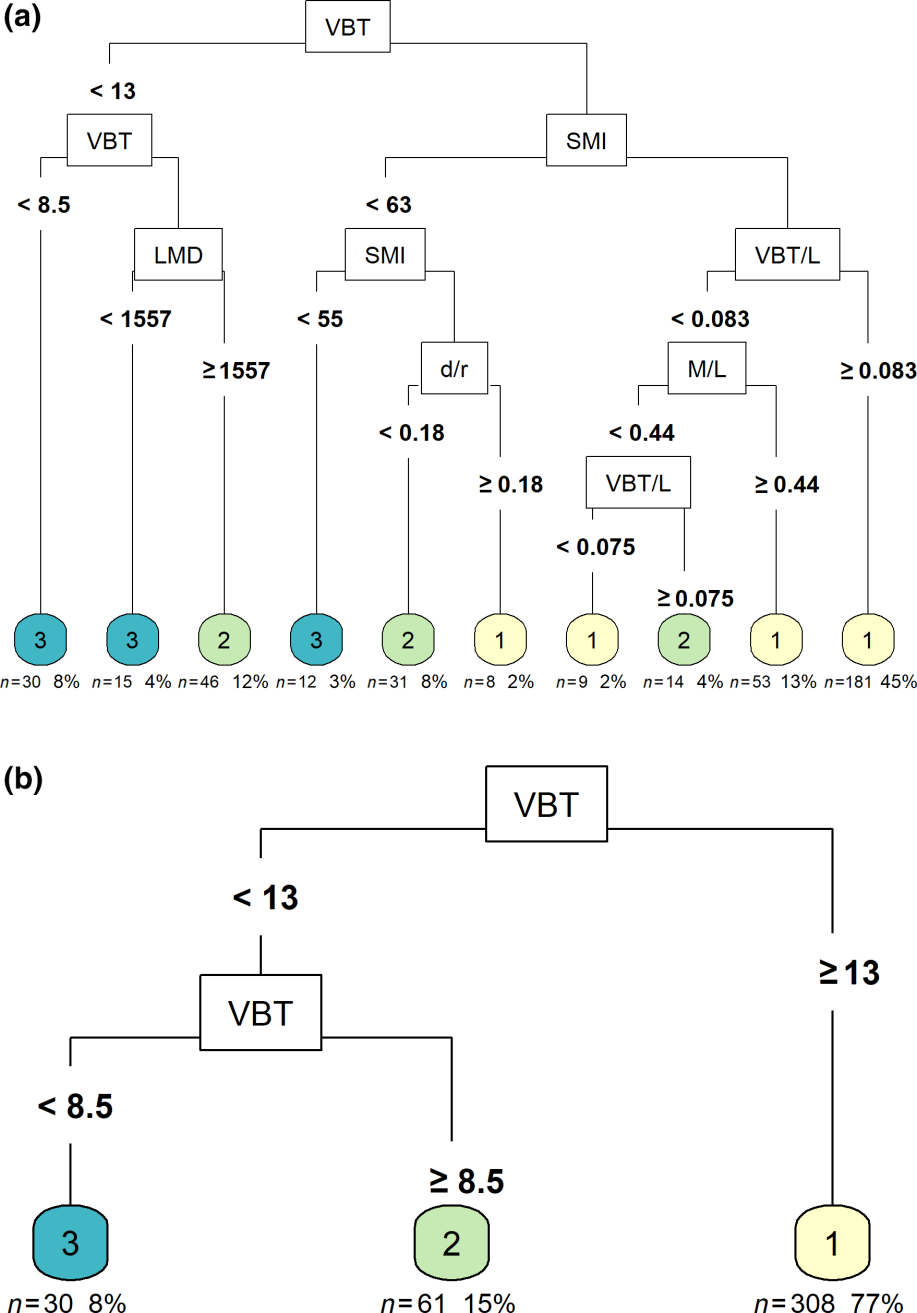
CART Trees for assessing the best predictor indices for Nutritional Status. (a) The optimal tree. (b) The reduced tree. The index that defined a split was labelled at each split along with break values labelled at the branch. Nutritional status classifications were at the terminal nodes, where good = 1, moderate = 2 and poor – very poor = 3. Below the nodes, the number and percentage of observations are displayed.

The optimal tree, obtaining the highest accuracy and pruned to the lowest misclassification cost, resulted in 10 terminal nodes (Appendix S1) and split data entries according to six variables: VBT, SMI, LMD, VBT/L, d/r, and M/L (Figure 3a). When predicting the 20% testing data set the tree correctly classified 79.31% of good nutritional status, 57.69% of moderate nutritional status, and 60.00% of poor to very poor nutritional status (Table 3). The average classification accuracy was 70.71% (CI 95%: 60.71%–79.43%) and predicted significantly more correct than would be obtained by classifying as the most likely class (No Information Rate, *p* < .01). The reduced tree resulted in three terminal nodes and classified these data only according to the variable VBT (Figure 3b). The average classification accuracy on unseen (testing) data of this reduced tree had an identical value of 70.71% (CI 95%: 60.71%–79.43%) though with differing percentages per status: 70.63% of correct classifications for good nutritional status, 57.14% for moderate nutritional status, and 72.73% for poor‐to‐very poor nutritional status (Table 3).

**TABLE 3:** Confusion matrix when predicting the 20% unseen testing dataset using the model obtained through the 80% training set, where (A) used the optimal tree model and (B) the reduced tree model.

Prediction	A reference			B reference		
	Good	Moderate	Poor – very poor	Good	Moderate	Poor – very poor
Good	46	7	2	50	5	0
Moderate	10	15	4	14	12	3
Poor – very poor	2	4	9	3	4	8

*Note*: The matrix displays the number of cases of each nutritional status.

The two CART models exhibited an average accuracy rate of around 71%. Analysis of the confusion matrices revealed that the models displayed a slight inclination towards false negatives, suggesting a cautious prediction approach where a more severe condition is predicted than what actually existed. The moderate class proved to be the most challenging for both models, while the extreme classes were identified with greater precision, consistent with their characteristics. The middle section stretches in between, with values on either end being more readily distinguishable. The full tree achieved a significantly higher rate of correct predictions while maintaining the same level of accuracy as the reduced tree, compared to the default selection of the most likely class.

**FIGURE 4:**
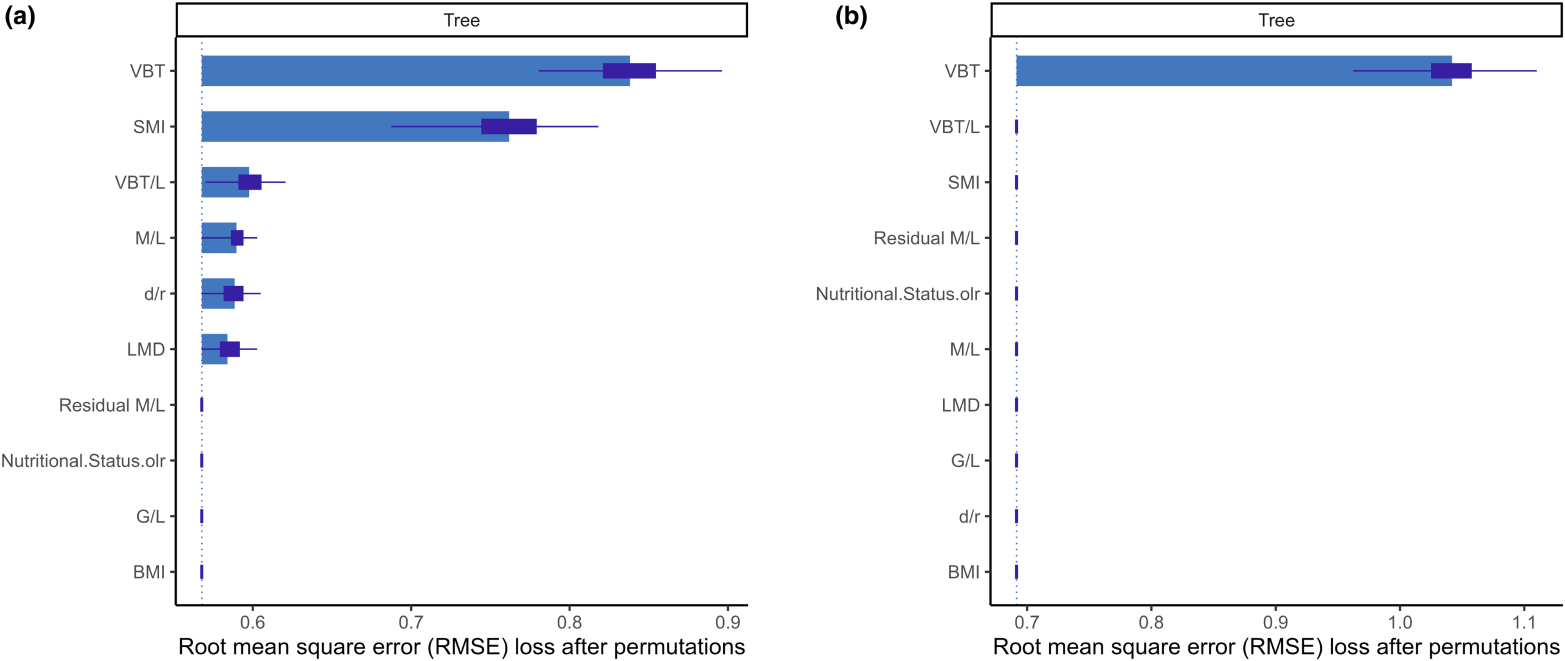
Variable importance plot for (a) all variables fed into the optimal tree model and (b) all variables of the reduced tree model.

VBT was found to be the most important predictor variable for both the optimal and reduced CART classification trees, followed by SMI for the optimal tree (Figure 4). Univariate partial dependence plots of the predictor variables for both trees displayed that the main predictor variable in both models was VBT (Appendix S1). Clear thresholds were observed, resulting in three plateaus that corresponded to a given nutritional status in contrast to other variables. Within the optimal model, the variable SMI demonstrated a comparable yet somewhat attenuated partial dependence curve, while variables BMI and Residual M/L also exhibited a plateau‐like configuration, albeit characterised by narrower deviations (Appendix S1). The remaining indices exhibited disorderly patterns of predictions. Similar to results observed in dependence plots for the reduced model for all indices, apart from VBT.

### 3.3 Variables influencing chosen body condition indices

#### 3.3.1 VBT – Variable selection

We undertook a generalised linear model (GLM) analysis to identify the main variables influencing nutritional condition, using VBT as the response variable. Individual causes of deaths, if sampled at the beach, or at sea and seasons spread across time and VBT, mirroring homogeneity of these data, despite the contemporary dataset only originating from stranded animals (Appendix S1). Residual diagnostic testing using DHARMa (bootstrap resampled 100 times) indicated no violations of model assumptions. The best fitting VBT model with the lowest AICc incorporated the response variables COD, time period (UK historical, IR contemporary), total body length, nutritional status, season, sex and sexual maturity status (AICc = 2259.66, *df* = 14, Appendix S1). Other models within two AICc units also included date reported as a significant variable. The model including all two‐dimensional interactions between the factors of the best‐fitting model did not result in an optimised fit (AICc = 2305.73, *df* = 71), and was therefore not explored further.

#### 3.3.2 VBT – Post hoc analyses

**FIGURE 5:**
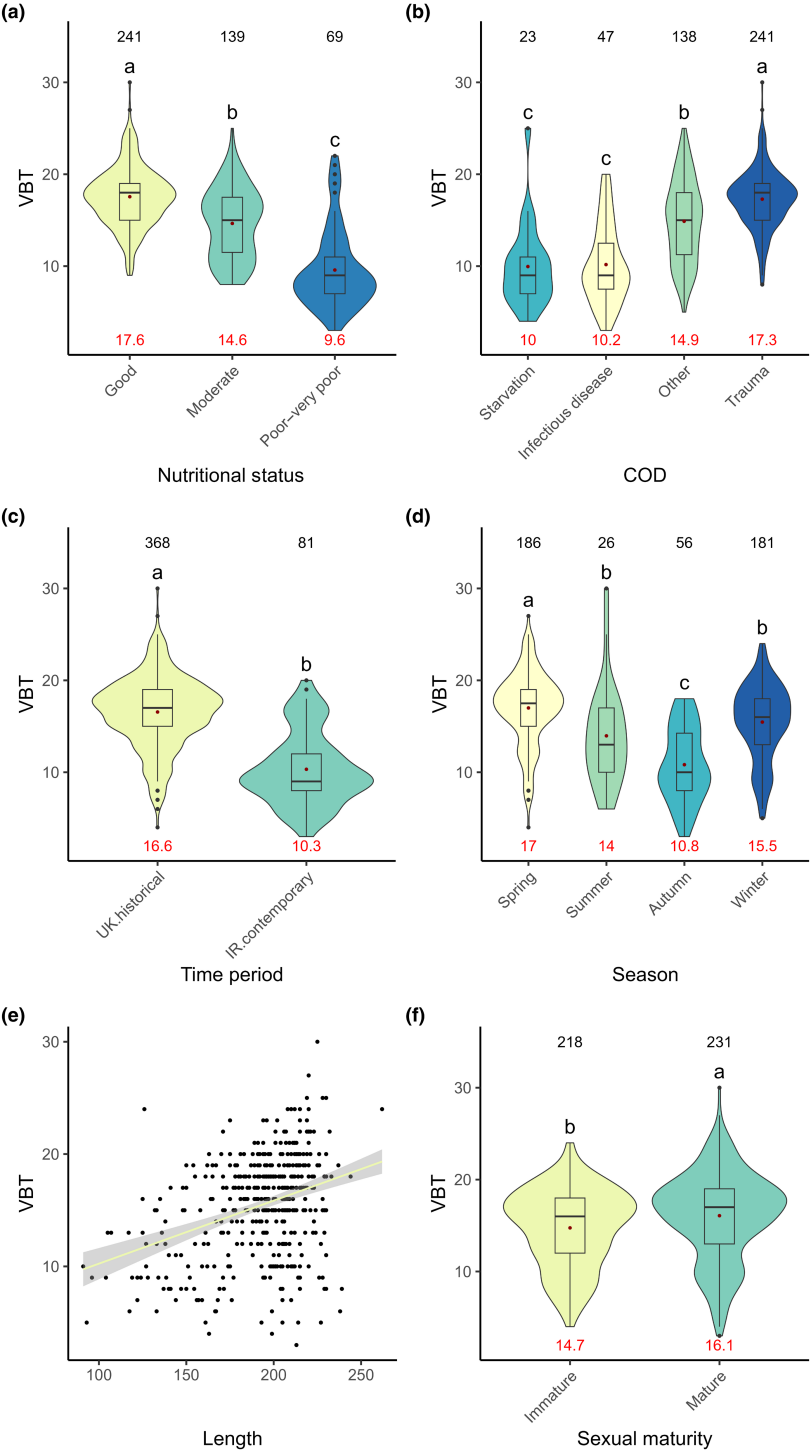
VBT (mm) across (a) Nutritional Status, (b) COD, (c) Time period, (d) Season, (e) Total body length (cm), and (f) Sexual maturity status. Significance is indicated through letters above the violins. Factor levels that significantly differ from each other display different letters. Number of observations is displayed on top of the graphs, the average value of the observations is indicated as a red dot within the plot, and the value is displayed at the bottom of each plot. In (e) a linear regression with 95% confidence intervals is plotted.

Using the optimal GLM model, significant differences were found between all categories of nutritional status based on the VBT model, as expected (*χ* ^2^ = 845.26, *p* < .001, Figure 5a). VBT was found to show a significant positive correlation with total body length (*χ* ^2^ = 552.78, *p* < .001, Figure 5e), and sexually mature individuals had significantly thicker VBT than sexually immature individuals (*χ* ^2^ = 33.02, *p* = .05, Figure 5f). Seasonal effects were also observed as VBT declined in relative thickness from spring to autumn (*χ* ^2^ = 351.10, *p* < .001, Figure 5d). ‘Starvation’ and ‘infectious disease’ cases had significantly thinner VBT when compared to ‘trauma’ cases, and individuals who died from ‘other’ causes of death (*χ* ^2^ = 2864.63, *p* < .001, Figure 5b). The Irish contemporary time period comprised individuals that had a significantly thinner VBT than the UK historical time period (*χ* ^2^ = 863.26, *p* < .001, Figure 5c). Although sex remained in the VBT model, no significant difference was observed between the sexes (*χ* ^2^ = 12.72, *p* = .23).

#### 3.3.3 SMI – Variable selection

When assessing the SMI model with DHARMa, no major issues were found in the distribution of the residuals, but some outliers were detected. After reviewing those entries, the outliers were considered as biological outliers and kept within the data set as they represented natural variation. The best‐fitting model of the SMI included predictors COD, total body length, nutritional status, and sexual maturity status (AICc = 2909.85, *df* = 9, Appendix S1). After including two‐dimensional interactions among variables, the model fit did not improve and the model was not explored further (AICc = 2915.31, *df* = 24). Season and time period were not observed to be significant predictors in the SMI model.

#### 3.3.4 SMI – Post hoc analyses

**FIGURE 6:**
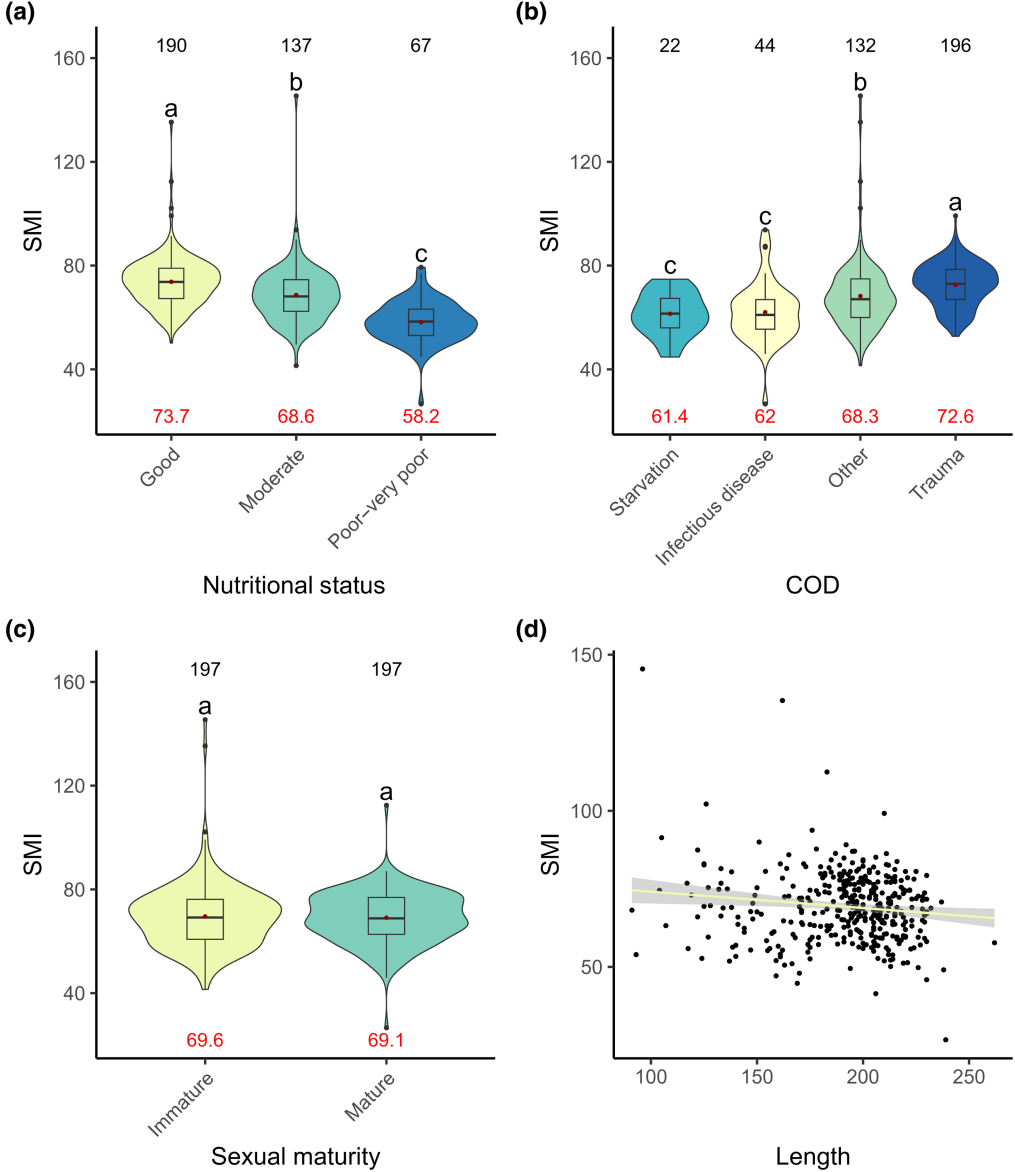
SMI across (a) Nutritional status, (b) COD, (c) Sexual maturity status, and (d) Total body length (cm). Significance is indicated through letters above the violins. Factor levels that significantly differ from each other display different letters. Number of observations are displayed on top of the graphs, the average value of the observations is indicated as a red dot within the plot, and the value is displayed at the bottom of each plot. Where are linear regression is fitted, 95% confidence intervals are displayed.

Like VBT, the SMI was found to be a good indicator of nutritional condition, as all nutritional status categories varied significantly, with the SMI observed to decrease with declining nutritional status (*χ* ^2^ = 6402.5, *p* < .001, Figure 6a). Further, individuals who died from ‘starvation’ and ‘infectious diseases’ had significantly lower SMIs than those who died from ‘trauma’ and ‘other’ causes of death (*χ* ^2^ = 5948.9, *p* < .001, Figure 6b). While, the parameter sexual maturity status was retained within the final model, no significant difference was observed between sexual maturity status categories (sexually immature vs. sexually mature) within the post hoc analysis (*χ* ^2^ = 1310.9, *p* < .001, Figure 6c). However, a significant negative correlation was found between total body length and SMI (*χ* ^2^ = 1720.4, *p* < .001, Figure 6d). Other models that were within two AICc units included time period.

### 3.4 Assessment of SMI applicability: Correlation with total body length

Upon further analysis, it was observed that the residuals of the standard major axis regression, which were calculated during the estimation of scaled mass index (SMI), shifted towards negative values for larger animals (Appendix S1). Additionally, it was found that these residuals were negatively correlated to the log‐transformed total body length of the animal (Pearson's product–moment correlation: log(total body length) ~ residuals(ft), *τ* = −3.0003, *p* < .001). Hence, the assumption that the relationship between the log‐transformed total body length and the residuals of the fit was linear was not met, at least not for larger‐sized animals.

## 4 DISCUSSION

A decline in the nutritional health of the common dolphin in the Celtic Seas ecoregion was observed between time periods assessed within the current study, with an increased number of stranded individuals presenting in an emaciated condition and dying as a result of starvation in the most recent time period, as well as a significant decline in overall nutritional status and VBT between time periods.

### 4.1 Suitability of morphometric body indices to predict nutritional status: VBT and SMI

#### 4.1.1 Ventral blubber thickness

VBT was identified to be the most important morphometric body condition index predicting the nutritional status of common dolphins within the current study. When identifying the importance of variables by measuring the effect of their removals, it ranked highest in the full model and exclusively explained the reduced model.

Previously, measures of blubber thickness have proven to be a suitable, straightforward yet effective tool for monitoring nutritional health in a range of marine mammal species and applications. Blubber thickness was recommended as a suitable metric to evaluate the nutritional condition of harbour seals, grey seals, and harbour porpoises in the Baltic Sea (Siebert et al., 2022). However, no other body condition index was considered within the study. Other work, for example, on North Atlantic fin whales identified a negative density dependent response in the pregnancy rate, through a decrease in blubber thickness resulting from reduced per capita prey availability (Williams et al., 2013). There has however been some debate on the appropriateness of blubber thickness as a condition metric (Derous et al., 2020), given the multifunctionality of blubber (Derous et al., 2020; Koopman, 1998; Noren & Wells, 2009). Additionally, blubber thickness is correlated with total body length, as observed in this and other studies (e.g., Gómez‐Campos et al., 2011; Siebert et al., 2022; Stevenson & Woods, 2006), which should be considered in assessments of nutritional condition. In odontocetes, obtaining blubber measurements within the thoracic region is important, as the blubber in these areas rather represent energy reserves than structural functioning, such as posterior regions of the animals (Koopman, 1998; Koopman et al., 2002). Above all, a standardised location for sampling is essential in order to create robust measures (Ijsseldijk et al., 2019; Noren et al., 2015, 2021; Ryan & Kershaw, 2022).

#### 4.1.2 Scaled mass index

The SMI was the second most important predictor variable in the full tree model and might therefore supplement the VBT index within indicator assessments. The SMI was originally recommended as a novel condition index being independent of body length which is important, given that a specific body size may require a specific optimum energy reserve (Peig & Green, 2009, 2010). Previously, the SMI was reported to be consistent when applied across different age‐sex groups, but results were inconsistent across geographic regions, possibly due to biological variations within a species (Peig & Green, 2010). Thus, care needs to be taken when comparing SMIs between studies. Applying the SMI to beluga whales (*Delphinapterus leucas*) proved effective for representing a visual qualitative evaluation of condition (Larrat & Lair, 2021), similar to the nutritional status of this study. Contrary to expectations, this applied only to larger‐sized beluga whales (>290 cm), while smaller animals posed challenges, indicating potential biases in the assumed power relationship between mass and length (Larrat & Lair, 2021). Our study further revealed the SMI's length dependency, but only for larger‐sized animals. The surface‐area‐to‐volume ratio of cetaceans decreases with age, which influences thermoregulation and buoyancy through blubber thickness with a larger area, resulting in a higher loss of heat (Noren & Wells, 2009). Younger bottlenose dolphins have been reported to increase their insulation by depositing more blubber relative to muscle mass than adults to minimise body heat and energy loss (Adamczak et al., 2021; McLellan et al., 2002). Potentially, to counteract heat loss effects at smaller body sizes, younger common dolphins were proportionally heavier within the current study. Although not fully length‐independent, the index might still be useful. However, it requires more resources in the field than measuring VBT, and VBT is ranked of higher importance for predicting nutritional status.

Other indices, such as the BMI (Kershaw et al., 2017) and Blubber Trunk Lipid Mass (BTLM) (Gómez‐Campos et al., 2011) previously outperformed measures of blubber thickness for other small cetacean species, such as harbour porpoise and striped dolphin, reflecting both species differences and methods of assessment.

### 4.2 Nutritional health of common dolphins in the NE Atlantic

#### 4.2.1 Biological and health‐related variations in nutritional condition

The VBT index model identified significant differences within categorical variables nutritional status, COD, time period, season, and sexual maturity status and also for the continuous variable, total body length. While significant differences between sexes were not detected in this moderately sexually dimorphic species (Murphy & Rogan, 2006), sex remained as a variable in the optimal model, as well as other models within two AICc units. Total body length and sexual maturity status were both significant predictors, though no collinearity was observed.

Animals that died due to ‘infectious disease’ and ‘starvation’ had thinner VBT, likely due to depleted energy reserves. Conversely, those who died from acute ‘trauma’ or ‘other’ causes had thicker blubber, indicating that this group likely consisted of healthy individuals, as previously reported for harbour porpoises in UK waters (Kershaw et al., 2017).

#### 4.2.2 Temporal variations in nutritional condition

Nutritional condition was observed to vary across seasons, being optimal in spring, and least favourable during the autumn, as indicated by seasonal fluctuations in VBT. These fluctuations are likely attributed to seasonal changes in prey availability, reproductive investment and thermoregulatory needs. Previously, offshore summertime movements and inshore wintertime movements in western European waters were suggested to occur due to the energy requirements of pregnant and lactating females and their calves (Brophy et al., 2009; Murphy et al., 2013). With individuals moving offshore during the summer when neritic prey is either nutrient poor (i.e., lower calorific value) due to spawning or has dispersed from spawning grounds, enabling individuals to take advantage of the higher lipid content myctophid's and juvenile horse mackerel (*Trachurus trachurus*) (Brophy et al., 2009; Murphy et al., 2013; Spitz et al., 2012). From October to March, they predominantly feed on moderate‐quality prey like *Trisopterus* sp., Gobidae sp., blue whiting (*Micromesistius poutassou*), and whiting (*Merlangius merlangus*) (Brophy et al., 2009; Spitz et al., 2010). During this period, their body condition improved, reaching its peak in spring, before declining again during the summertime mating and calving seasons.

Congruently, thermoregulatory processes might also drive seasonal variations in blubber thickness. Seasonal sea surface temperatures in Irish waters can vary by 6–7°C, on average; ranging from 9 to 10°C, on average, in February, March and April (spring in the current study), to 15–16°C, on average, in July, August and September (summer/autumn in the current study) (Casal & Lavender, 2017). Common dolphins in the NE Atlantic are likely to adjust their blubber thickness because of thermoregulation according to those seasonal fluctuations, similar to bottlenose dolphins in the West Atlantic (Adamczak et al., 2021; Meagher et al., 2008). Therefore, sexually immature juveniles also show seasonal fluctuations in ventral blubber thickness.

VBT decreased by approximately 30% during the study period. Despite a 0.2–0.3°C per decade increase in NE Atlantic sea surface temperatures (Diez et al., 2022), this change is minor compared to seasonal variations common dolphins adapt to in Irish waters (Casal & Lavender, 2017). As Murphy et al. (2009) found no significant difference in the nutritional status of the NE Atlantic population between the 1990s and early‐to‐mid 2000s, until further analysis is undertaken, it remains hard to decipher what effect a further 0.2–0.3°C increase in sea surface temperature may have on thermoregulation in this highly mobile marine species. Additionally, the average sea temperature at 0–200 m depth range has largely plateaued since 2005 (Diez et al., 2022), and the final GLM for VBT did not include the date variable, though it appeared in other models within two AICc units. Therefore, temperature may not have been the primary cause of the decline in nutritional health bewteen time periods. However, the indirect effects of varying sea temperatures through altering prey availability require further investigation.

#### 4.2.3 Ecological perspective

While recent management efforts have curbed some overfishing in the NE Atlantic (Hilborn et al., 2020), specific fish stocks, such as herring (*Clupea harengus*), are still facing challenges due to overexploitation (ICES, 2023), while monitoring gaps exist for other prey species of the common dolphin, like sprat. Environmental change‐induced alterations, such as the increasing presence of European anchovy (*Engraulis encrasicolus*), as observed in Irish groundfish surveys, signify ecosystem shifts (Vaughan et al., 2023). These shifting ecosystem dynamics may likely have impacted the nutritional health of common dolphins, in addition to the depletion of fish stocks due to overfishing. The decline in herring populations influenced the foraging behaviour of common dolphins in the Celtic Sea, south of Ireland during autumn, where the presence of common dolphins was associated with herring abundance (Fariñas‐Bermejo et al., 2023). However, during summer surveys at the Malin Shelf, north of Ireland, common dolphin presence was not correlated with herring or horse mackerel biomass but with substrate type, indicating potential variations in feeding ecology between locations, with dolphins in the north possibly relying more on demersal fish communities (Pommier et al., 2023). These complexities highlight the challenges in predicting the effects of ecosystem shifts on common dolphin populations. The commercial exploitation of cephalopods presents a unique challenge due to limited data and their rapid life cycle, and lack of monitoring under the EU Common Fisheries Policy (Arkhipkin et al., 2021). Cephalopod species are likely to excel in ecological niches left unoccupied by other species and are projected to increase in biomass as ocean conditions shift (Arkhipkin et al., 2021). Evidence suggests a dietary shift in sea bird top predators from fish to cephalopods due to changing prey communities (Gagne et al., 2018), a shift potentially mirrored by other top predators, such as common dolphins, which requires further investigation.

While the nutritional health of common dolphins may be declining, at least on a regional level, within the NE Atlantic population, the effects of this have not been observed at the population level. Large‐scale surveys undertaken over a 17‐year period reported abundance estimates of 468,400 (CV = 33%) (surveys 2005/2007), 487,100 (CV = 25%) (survey 2016) and 439,212 (CV = 18%) (survey 2022) common dolphins in continental self and adjacent waters of the NE Atlantic (Geelhoed et al., 2022; Gilles et al., 2023). The extent of those surveys did not cover the full (unknown) range of the NE Atlantic population, and the latter abundance estimate excluded Irish waters – for which the results are still pending. Further, the abundance estimates were deemed as minimum estimates, due to the observation of other dolphins that were not identified as species (categorised as unidentified common/striped dolphins). While it may take a longer period of time before the impacts of a stressor, such as nutritional stress, can be detected at the population level, shifts in their distribution have been reported. Common dolphins have shown long‐term distributional shifts in response to changes in pelagic ecosystems, possibly to remain within their ecological niche (Murphy et al., 2013). More recent shifts in the main occurrence of the species in the Bay of Biscay have also been observed, suggested to be related to varying prey abundance (Astarloa et al., 2021), as well as a northwards expansion in the NE Atlantic (Plint et al., 2023).

In other cetaceans, changes in prey availability have been shown to affect fitness and reproduction. For instance, fluctuations in Antarctic krill (*Euphausia superba*) led to a decline in maternal body conditions and reproductive success in Southern right whales (Vermeulen et al., 2023). As noted earlier, Atlantic fin whales also experienced reduced body conditions due to lower per capita plankton biomass, leading to a lower probability of pregnancy (Williams et al., 2013). Thus, the quantity of prey may be more important than its quality for filter feeders like mysticetes. Whereas, for odontocetes, like the harbour porpoise, prey quality was vital for maintaining body condition (Leopold et al., 2015) and achieving reproductive success. Pregnancy rates in harbour porpoises were observed to be positively correlated with prey energy densities, while females in a poorer health status had a lower probability of being pregnant and of a successful pregnancy (Ijsseldijk et al., 2021).

Nutritional constraints were suggested as the primary cause for the decline in the Steller sea lion population in the Gulf of Alaska and Aleutian Islands (Donnelly et al., 2003; Rosen & Trites, 2005; Trites & Donnelly, 2003). Chronic nutritional stress, caused by lower quality of prey (rather than insufficient quantity) led to a reduction in body size and biochemical blubber composition (Donnelly et al., 2003; Rosen, 2009; Rosen & Trites, 2000, 2005; Trites, 2021; Trites & Donnelly, 2003). Additionally, a decrease in the birth frequency was observed, with extended nursing periods for pups beyond 1 year (Rosen, 2009; Trites, 2021; Trites et al., 2006). The chronic nutritional stress observed stemmed from a decline in prey rich in fats (Rosen & Trites, 2000; Trites & Donnelly, 2003). Although experimental studies on Steller sea lion had certain biases, behavioural studies generally supported the junk‐food hypothesis rather than the overfishing/ abundance hypothesis (Trites, 2021). Other pinnipeds, such as the California sea lion (*Zalophus californianus*), also suffered from nutritional stress due to lower‐quality food resulting from prey shifts, which was evident in their low reproductive success and pup survival, underlining the importance of addressing long‐term environmental shifts altering prey composition for marine predator conservation efforts (McClatchie et al., 2016).

### 4.3 Caveats

#### 4.3.1 Mitigation of sampling biases

Establishing a reliable marine mammal population condition indicator presents challenges due to the need for long‐term standardised data sets obtained through long‐term health and necropsy monitoring programmes, which are not restricted to a particular geographic area (Siebert et al., 2022). Within the current study, we utilised data from monitoring programmes that spanned several decades and followed standardised protocols for post‐mortem examinations. Unlike other small cetaceans, such as the harbour porpoise, stranded and bycaught common dolphins were previously found to be generally in good overall health and nutritional status, and for stranded animals the primary cause of death was largely attributed to interactions with fishing gear (Bennett et al., 2000; Deaville & Jepson, 2011; Sabin et al., 2005; SACVSD, 2000). The contemporary sample set was comprised of common dolphins that were stranded along the Irish coast between 2017 and 2019, with one of the project's primary aims to assess for evidence of fisheries interactions (Levesque et al., 2021). Thus, it was not assumed that a strong bias towards sampling ‘unhealthy’ stranded animals, such as animals in poor nutritional health, occurred during the contemporary period compared to previously. All datasets incorporated animals that died of trauma, assumed to represent a ‘healthy’ subsample. Further, the inclusion of parameters ‘causes of death’ and ‘nutritional status’ within our generalised linear models underscores the impartiality of our findings. Animals originating from all COD categories were spread across all sampling periods. While seasonality may partially drive the observed variability in VBT, animals were sampled throughout the year to account for this. However, it must be noted that the contemporary data set was comprised of relatively fewer animals, with fewer stranded animals that died as a result of trauma. Analysis of contemporary UK data may fill this sampling gap, as well as continued sampling of stranded animals on Irish coastlines.

#### 4.3.2 Model validation and bias mitigation: Ensuring robustness in analytical frameworks

The CART model performance highlighted the effectiveness of the optimal tree. However, it is important to note that such tree models are susceptible to overfitting (Therneau & Atkinson, 1997), which is why it was prudent to also evaluate the reduced tree. Although its performance did not differ significantly, it is possible that the disparity in sample size may have played a role. Upon examination of the variable importance plots and the root mean square error (RMSE) loss, it became clear that both decision trees yielded the same biologically relevant outcome.

We employed a generalised linear model within the current study. Among the models we tested for VBT, the one with the lowest AICc score was found to be significant and accurately identified the relevant variables and dynamics. Moreover, we confirmed that all the model assumptions were valid using the DHARMa tool (Hartig, 2022). Previous critiques of model selection based on the lowest AICc, such as that used in the dredge function, noted that it can result in spurious correlations by allowing the model to determine influential factors, rather than selecting them based on biological reasoning (Burnham & Anderson, 2003). However, all variables included in the models were biologically relevant in our case.

Another potential drawback of using model selection with the dredge function is that data must be reduced to full cases before analysis, which resulted in a smaller sample size and the exclusion of the Irish historical dataset. Furthermore, it would have been advantageous to select from all global models that include all possible interactions, but this was not feasible due to the limited number of models that can be computed within dredge. Therefore, only interactions between significant factors were incorporated. Although it is possible that other interactions may have played a role, AICcs were not found to improve with the inclusion of interactions among significant factors.

## 5 CONCLUSIONS

The nutritional health of free‐ranging animals is of growing interest in conservation research, being directly related to an individual's survival and reproductive success (Kershaw et al., 2017; Larrat & Lair, 2021; MacLeod et al., 2007; Murphy, 2015; Negri et al., 2014). For conservation management of a species, it is crucial to identify problems at an early stage. Here, we identified a decline in the nutritional health of one of the most abundant top predators in the NE Atlantic and developed a population condition indicator suitable for monitoring purposes and reporting under the the EU MSFD and by OSPAR. The current study offers a novel and more powerful approach for the evaluation of morphometric indicators than previously, by using ordinal regression tree analysis and including all the indices within one model instead of comparing single model outputs. The model was specifically designed to determine how important each index was for explaining nutritional status, using machine learning.

Despite the debate on employing measures of blubber thickness as a viable body condition metric, our results advocate the use of VBT for the common dolphin in the NE Atlantic, as it was found to reliably indicate an animal's nutritional status. VBT is a feasible parameter to measure in the field by both stranding and fishery observer programmes, with little equipment and training required. However, it is necessary to consider the effects of other variables, such as season and total body length, within any indicator assessments. The SMI could be considered as a supporting measure.

To better understand impending shifts in population dynamics, an updated assessment of temporal changes in parameters that exhibit density dependent compensatory responses should be undertaken at a population level. Assessing parameters such as juvenile survival, age at sexual maturity and pregnancy rate (Fowler, 1984; Lockyer, 1990; Murphy et al., 2009), as well as other fitness‐related traits. Additionally, investigating age‐length dynamics across time periods would be crucial in unveiling any temporal body size‐related variation. Molecular studies focused on metabolism could also provide additional insights into the phenomenon. Additionally, further research on dietary consumption and preferences of the contemporary sample set may uncover the root causes of the observed decline in the nutritional health of these individuals, by assessing the quantity and quality of prey consumed across nutritional status categories. Nutritional stress however is not the only threat and pressure impacting the NE Atlantic common dolphin population (Gosnell et al., 2024; ICES, 2020; Murphy et al., 2013, 2018, 2019), and an understanding of the complex nature of interactions between stressors is required.

## AUTHOR CONTRIBUTIONS

**Sofia Albrecht:** Conceptualization (lead); data curation (lead); formal analysis (lead); funding acquisition (lead); investigation (lead); methodology (lead); project administration (lead); resources (lead); software (lead); validation (lead); visualization (lead); writing – original draft (lead); writing – review and editing (lead). **Cóilín Minto:** Conceptualization (lead); data curation (lead); formal analysis (equal); funding acquisition (equal); investigation (lead); methodology (lead); supervision (equal); validation (equal); writing – original draft (equal); writing – review and editing (equal). **Emer Rogan:** Data curation (equal); resources (equal); writing – review and editing (equal). **Rob Deaville:** Data curation (equal); project administration (equal); resources (equal); writing – review and editing (equal). **Jim O'Donovan:** Data curation (equal); resources (equal); writing – review and editing (equal). **Mags Daly:** Data curation (equal); resources (equal); writing – review and editing (equal). **Stephanie Levesque:** Data curation (equal); resources (equal); writing – review and editing (equal). **Simon Berrow:** Data curation (equal); resources (equal); writing – review and editing (equal). **Andrew Brownlow:** Data curation (equal); resources (equal); writing – review and editing (equal). **Nicholas J. Davison:** Data curation (equal); resources (equal); writing – review and editing (equal). **Orla Slattery:** Funding acquisition (equal); project administration (equal); resources (equal); supervision (equal); writing – review and editing (equal). **Luca Mirimin:** Funding acquisition (equal); project administration (equal); resources (equal); supervision (equal); writing – review and editing (equal). **Sinéad Murphy:** Conceptualization (lead); data curation (lead); formal analysis (equal); funding acquisition (equal); investigation (lead); methodology (lead); project administration (equal); supervision (lead); validation (equal); writing – original draft (equal); writing – review and editing (equal).

## FUNDING INFORMATION

This study constitutes a component of the broader project titled “Impacts of anthropogenic activities and environmental change on the foraging ecology and nutritional status of common dolphin and its implications towards sustainable resource management,” funded through the Irish Research Council under the grant GOIPG/2021/378. The remaining authors did not secure specific funding specifically earmarked for this study. The data in this study were supported by funding from prior projects, only being provided for use in the present investigation: For the Irish contemporary dataset, dolphins were collected for post‐mortem under the Marine Institute‐EMFF funded Irish Vertebrate Necropsy Project (2017–2019), awarded to the Irish Whale and Dolphin Group, the Cork Regional Veterinary Laboratory and the Atlantic Technological University (formerly Galway‐Mayo Institute of Technology) (Tender Reference Numbers: ITT17‐024, ITT18‐005, and ITT18‐050). For Irish historical dataset, grants were awarded to University College Cork from the Heritage Council, National Parks and Wildlife Service, INTERREG Ireland – Wales, and two EC funded projects: FAIR‐CT95‐0523: Assessment and Reduction of the By‐catch of Small Cetaceans of Small Cetaceans (BY‐CARE) and EVK3‐2000‐00027: Bioaccumulation of persistent organic pollutants in small cetaceans in European waters: transport pathways and impact on reproduction (BIOCET). The collaborative Cetacean Strandings Investigation Programme (http://ukstrandings.org/) is funded by the Department for Environment, Food and Rural Affairs (Defra) and the Devolved Welsh Government. The Scottish Marine Animal Strandings Scheme is funded by the Devolved Scottish Government.

## CONFLICT OF INTEREST STATEMENT

The authors declare that the research was conducted in the absence of any commercial or financial relationships that could be construed as a potential conflict of interest.

## Supporting information


Appendix S1

## Data Availability

The data employed in this research emerged through long‐term monitoring projects and is governed by disclosure provisions outlined in collaborative agreements with ATU, the Marine Institute (https://www.marine.ie/) and the Cetacean Strandings Investigation Programme (CSIP, https://ukstrandings.org/). Access to the datasets can be applied through the Cetacean Strandings Investigation Programme (CSIP) archive for the UK dataset and via ATU, UCC and the Marine Institute data archive for the Irish datasets. We provide a randomly generated example data file to illustrate our data and increase transparency together with the R scripts and a metadata file at https://doi.org/10.5281/zenodo.11221584.
