# Supplementary material for: Emaciated enigma: Decline in body conditions of common dolphins in the Celtic Seas ecoregion
Source: Ecol Evol. 2024 Sep 30;14(10):e70325. doi: 10.1002/ece3.70325 (PMC11442009; doi:10.1002/ece3.70325)
Supplement: Supplementary file 1 — Appendix S1 [file ECE3-14-e70325-s001.pdf]

Supporting Information for:

Emaciated enigma: Decline in body conditions of common dolphins in the Celtic Seas ecoregion

Sofia Albrecht, Cóilín Minto, Emer Rogan, Rob Deaville, Jim O'Donovan, Mags Daly, Stephanie Levesque, Simon Berrow, Andrew Brownlow, Nicholas J. Davison, Orla Slattery, Luca Mirimin and Sinéad Murphy

Table of Contents:

**Table S1: Body index literature review containing references, experimental set up, the indices and their formulas, as well as the outcomes of the studies.**	Page 2
**Table S2: Models with VBT as response variable with AIC within the lowest 2 units obtained through dredge.**	Page 11
**Table S3: Models with SMI as response variable with AIC within the lowest 2 units obtained through dredge.**	Page 11
**Figure S1: Complexity parameters of the optimal tree.**	Page 12
**Figure S2: Univariate partial dependence plots of A) all variables and their prediction using the optimal model and B) using the reduced model.**	Page 13
**Figure S3: Ventral blubber thickness (mm) from 1990 to 2019 per A and C) All data, B and D) Irish historical and contemporary, only. Sample points were colour coded after A and B) causes of death and C and D) A general additive model smoothed curve is displayed with 95% confidence intervals.**	Page 14
**Figure S4: Ventral blubber thickness (mm) from 1990 to 2019 per A) All data, B) Irish historical and contemporary, only. Sample points were colour coded after seasons. A general additive model smoothed curve is displayed with 95% confidence intervals.**	Page 14
**Figure S5&6: VBT (mm) across seasons per data set.**	Page 15
**Figure S7: Residuals of the SMA regression of mass against body length (cm) populated during the calculation of the SMI.**	Page 16

Table S1: Body index literature review containing references, experimental set up, the indices and their formulas, as well as the outcomes of the studies.

	Experimental setup	Index	Units/Formula	Recommended index
**Harbour Porpoise**				
UK (Murphy 2015)	N = 329 females in fresh-to-moderate decomposition state<br>Indices and their residuals tested using six reproductive status categories: (1) neonates (<90 cm), (2) immature, (3), resting (non-pregnant, non-lactating), (4) lactating, (5) pregnant and (6) pregnant & lactating	Blubber lipid content<br><br>Ventral blubber thickness (front of dorsal fin)<br><br>Survival index<br><br>LMD-index<br><br>d/r ratio	% lipid<br><br>(mm)<br><br>$\frac{Body\ length}{Girth}$<br><br>$\sqrt{\frac{Body\ length}{Body\ weight}} \times Blubber\ tickness \times 100$<br><br>$\frac{Blubber\ thickness}{Girth}$	None deemed suitable due to experimental setup, and employing different reproductive status categories
Scotland/ UK (Kershaw et al., 2017)	N = 291 individuals<br>Each Index were modelled using a generalized linear model against the cause of death categories (1) acute cases assumingly representing good body conditions and (2) chronic cases representing emaciated animals, age class and season. The dataset was therefore split between maturity stages and breeding and non-breeding animals	Ventral blubber thickness<br><br>Ventral blubber:girth<br><br>Ventral blubber:body length<br><br>Girth:body length	(mm)<br><br>$\frac{Full\ ventral\ blubber\ depth}{Girth}$<br><br>$\frac{Full\ ventral\ blubber\ depth}{Body\ length}$<br><br>$\frac{Girth}{Body\ length}$	The Quetelet's index (BMI = mass/body length^2) resulted in the best model fit and therefore was recommended for future use.

		Mass:body length<br><br>Fulton's index K<br><br>Quetelet's index BMI<br><br>Relative condition Kn<br><br><br><br><br><br>Residual index Ri<br><br><br>Scaled mass index	$\frac{Mass}{Body\ length}$<br><br>$K = \frac{Mass}{Body\ length^3}$<br><br>$BMI = \frac{Mass}{Body\ length^2}$<br><br>$Kn = \frac{Mi}{Mi^*}$<br>Where Mi represents the individual mass and Mi* the predicted mass $Mi^* = aL_i^b$ where a and b are determined by ordinary least squares (OLS) regression of Mass against Body length for the whole study population<br><br>The residuals from an OLS regression of Mass against Body length, after log transformation<br><br>$\widehat{Mi} = Mi \times \frac{L0}{Li}^{bsma}$<br><br>where Mi is the mass of an individual, Li is its Body length, L0 is an arbitrary fixed body length and bSMA is the slope coefficient estimated from an SMA regression (Peig and Green, 2009)	
USA/ Canada (Koopman et al., 2002)	N = 100<br>Blubber thickness and adipocytes measurements were compared	Blubber thickness	(mm)	Blubber thickness close to the thorax but not to

	between healthy and starved animals using paired t-tests.			the tail is recommended.
West Greenland (Heide-Jørgensen et al., 2011)	N = 192<br>LMD and MC/L indices were compared between 2 years separated for male and female using t-test	LMD index<br><br>MC/L index	$\sqrt{Body\ length\ \times Mass}\ \times Blubber\ thickness$<br><br>$\frac{Girth}{Body\ length}$	Indices are applied without previous verification
Netherlands (IJsseldijk et al., 2021)	N = 199<br>Mature females were assessed after two cause of death categories: (1) acute and (2) debilitated cases. The data was corrected for season using a generalized additive model (GAM) and residuals were used for further analysis with PCB chemical pollutants and reproductive success. Nutritional condition codes (good, moderate, poor) was included in the further analysis	Mean blubber thickness	Average of three measures in a dorsoventral line on the left body flank just cranial to the dorsal fin, at three locations: dorsal, lateral, and ventral	Indices are applied without previous verification. Maternal nutritional and health status affected foetus size and the probability of being pregnant
UK (Williams et al., 2020)	N = 814<br>Residuals of a power regression model of weight to body length of the animals was used as nutrtitional body condition proxy with animals below the regression representing poor body condition and animals above the regression line representing good nutritional condition.<br>The nutritional body condition proxy was used to standardise the rest of the data set for an analysis of PCB concentration in the blubber and its relation to cause of death, age class,	Weight to body length ratio	$\frac{Mass}{Length}$	Index was applied without previous verification.<br>It was found that nutritional condition is related to death from infectious disease.

	season, and sex. Data was categorized per age class (subadults and adults) established through body body length and sexual maturity, per cause of death (trauma, infectious disease, other), and per season (winter, spring, summer, autumn) for further analysis.			
UK (Williams et al., 2021)	N = 267<br>A power regression model was fitted with body weight and body length and residuals were extracted as a proxy for body condition.<br>A linear mixed model (LMMs) was then fitted to understand the relation between mean testes weight, PCB blubber concentrations, nutritional condition and breeding season	Weight to body length ratio	$\frac{Mass}{Body\ length}$	Index was applied without previous verification.<br>PCBs exposures are associated with reduced testes weights in adults with good body condition.<br>In animals with poor body condition, PCBs impacted testes weights reduced, conceivably due to testes weights being limited by nutritional stress
Baltic Sea: Germany, Denmark and Poland (Siebert et al., 2022)	N = 345 individuals<br>Body conditions in the area were examined using blubber thickness as an indicator of energy reserves for reporting to the Marine Strategy Framework Directive.<br>A generalized additive model was applied using mean blubber thickness	Mean blubber thickness	Average of dorsal, lateral and ventral blubber thickness cranially to the pectoral fin, at the dorsal fin and caudally to the dorsal fin	Blubber thickness thickness is an interesting metric in a wider system of indicators, but not solely

	as a response variable and sex, age, day of the year and year			
**Common dolphin**				
USA (Joblon et al., 2014)	N = 30 individuals<br>A Body Scoring System was established based on visual features of the animals. This system was then tested grouping individuals by the type of stranding event: (1) mass stranded, (2) single stranded, (3) bycaught. Differences in indices where tested between those groups using Kruskal Wallis H test and a non-parametric ANOVA correcting for cofounding factors such as sex, age and season	Girth<br><br>Body length to girth ratio<br><br>Blubber thickness	(cm)<br><br>$L:G\ ratio = \frac{Body\ length}{Girth}$<br><br>(cm)	L:G ratios were not approved suitable as an indicator, but the verification of body indices is recommended for future research
**Bottlenose dolphin**				
USA (Hart et al., 2013)	N = 160 individuals<br>Indices were evaluated using stranded emaciated animals previous to their application. Then they were applied separate for sex, pregnant females were excluded, only summer measures were used to account for seasonality. Nonlinear and linear quantile regression methods were used to estimate the parameters for the reference ranges for the species	Ordinary least square models<br><br>Model 1:<br><br>Model 2:<br><br>BMI<br><br>Of those equations they calculate 95% confidence intervals. Animals below this intervals are then assumed to be in bad nutritional health in the future	$Mass = 10^a \times Body\ length^b$<br><br>$Girth = a + b \times Body\ length$<br><br>where a and b are estimated parameters<br><br>$BMI = \frac{Mass}{Body\ length^b} \times 10000$	All indices were applied and reference ranges were established

**Beluga whale**				
Canada (Larrat and Lair, 2021)	N = 236 individuals<br>The index was tested using a linear regression of an analog-visual scale of good body conditions vs emaciated body conditions and individuals were grouped in size classes: (1) >290cm body body length, (2) <290cm body body length	Scaled mass index $\widehat{Mi}$	$\widehat{Mi} = Mi \times \frac{L0}{Li}^{bsma}$<br><br>where Mi is the mass of an individual, Li is its body length, L0 is an arbitrary fixed body length and bSMA is the slope coefficient estimated from an SMA regression (Peig and Green, 2009)	The scaled mass index was deemed suitable for individuals larger than 290 cm body body length
**Franciscana dolphin**				
Argentina (Negri et al., 2014)	N = 54 individuals<br>Each index was applied between sexes, maturity class and area and differences between individuals were tested using Chi^2 , t-test and Mann–Whitney U-tests	Relative body condition index Kn<br><br><br><br><br><br><br>Fat index<br><br><br><br><br><br>liver index	$Kn = \frac{Wo}{We}$<br>where Wo is the measured total wet weight of the animal (kg) and the We is the total weight estimated (kg) from a standard body length–weight potential regression.<br><br>$Fat\ index = \frac{Wf}{Wo} \times 100$<br>where Wf is the weight of the fat in the blubber and Wo is the total body mass<br><br>$Liver\ index = \frac{Wl}{Wo} \times\ 100$<br>Where Wl is the weight of the liver and Wo is the total body mass	The liver index is recommended as a nutritional status indicator and baseline measures had been established for all indices.
**Striped dolphin**				
Spain (Gómez-Campos et al., 2011)	N = 115 individuals<br>The dataset was separated by sex and reproductive condition an categorized after:<br>1)calves/neonates	Blubber lipid content<br><br>Blubber Trunk Lipid Mass (BTLM)	% Lipid<br><br>$BTLM = \%\ Lipids\ in\ blubber \times Blubber\ mass$	BTLM was most accurate measure, followed by blubber lipid

	2) immatures<br>3)pregnant females<br>4)lactating females<br>5)resting females<br>6)mature males<br><br>Then they did a regression of the indices to body size, and the residuals of those regressions were used as indices of condition. Residuals were then compared between categories using ANOVA and tukey HSD	Muscle/blubber trunk mass ratio (M/B ratio),<br><br>Maximum girth<br><br>Blubber thickness	$\frac{M}{B} ratio = \frac{Muscle\ mass}{Blubber\ mass}$<br><br>(cm)<br><br>(cm)	content. M/B ratio, maximum girth and blubber thickness failed to reflect nutritional conditions of the animals.
**Rough-toothed dolphin**				
USA (Karns et al., 2019)	N = 68 individuals<br>Individual dolphins of two stranding events were compared through the study. Comparison of gender, age class, body condition, and outcome was compared between stranding events using a chi-square test. A general linear model (GLM) was used to compare mean BMI for each stranding event and control for differences in gender and age class. A GLM was used to compare BMI by outcome (natural death, euthanasia, release) while adjusting for age class and gender.	Body mass index	$BMI = \frac{Mass}{Length}$	Body mass index
**Review analysis**				
(Castrillon and Bengtson Nash, 2020)	Review article on cetacean body condition evaluations	Girth<br><br>girth body length	(cm)<br><br>$\frac{Girth}{Length}$	Girth was found to be sometimes useful, sometimes not,

		other measures not detailed		depending on species and reproductive status<br><br>Girth-body length: was found to function well as an indicator of body conditions, but it is species and age category specific and reflects anomalies of growing individuals<br><br>Any index calculated is necessary to be considered in relation to non-morphological parameters that are known to influence blubber variation, such as sex, age, reproductive class/state, day in the feeding season, and stage of the annual re-

				productive cycle to improve the indices' accuracy.
(Derous et al., 2020)	Review on markers of health of cetaceans	Blubber thickness	(cm)	Blubber thickness alone is not an accurate measure to identify body condition. It is recommended to conduct interdisciplinary analysis including energy metabolism pathways

Table S2: Models with VBT as response variable with AIC within the lowest 2 units obtained through dredge.

Model	Variables	df	AICc	Delta AICc
1	COD + Data source + Body length + Nutritional Status + season + Sex + Sexual Maturity	14	2259.66	0
2	COD + Data.source + Date reported + Body length + Nutritional Status + season + Sex + Sexual Maturity	15	2260.39	0.72
3	COD + Data source + Body length + Nutritional Status + season + Sexual Maturity	13	2260.77	1.11
4	COD + Data source + Body length + Nutritional Status + season	12	2260.81	1.15
5	COD + Data source + Body length + Nutritional Status + season + Sex	13	2261.44	1.77

Table S3: Models with SMI as response variable with AIC within the lowest 2 units obtained through dredge.

Model	Variables	df	AICc	Delta AICc
1	COD + Body length + Nutritional status + Sexual Maturity	9	2909.85	0
2	Body length + Nutritional status + Sexual Maturity	6	2911.40	1.56
3	COD + Data source + Body length + Nutritional status + Sexual Maturity	10	2911.54	1.69

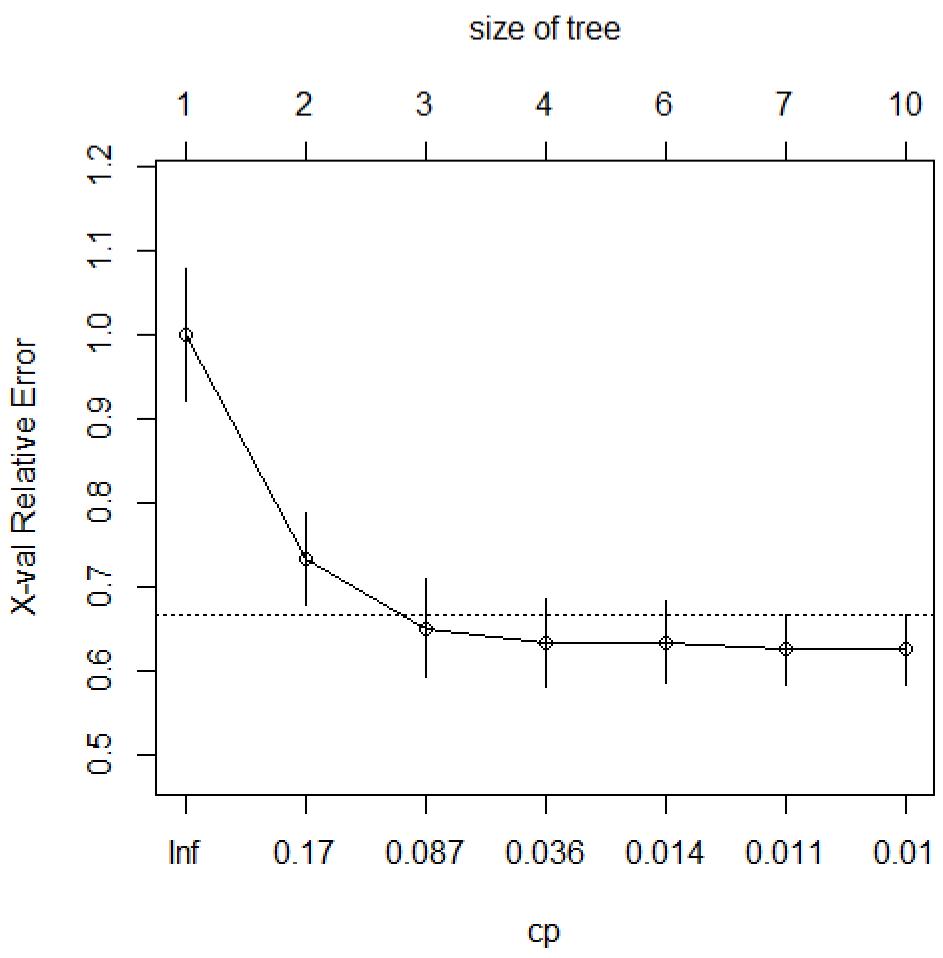

Figure S1: Complexity parameters of the optimal tree.

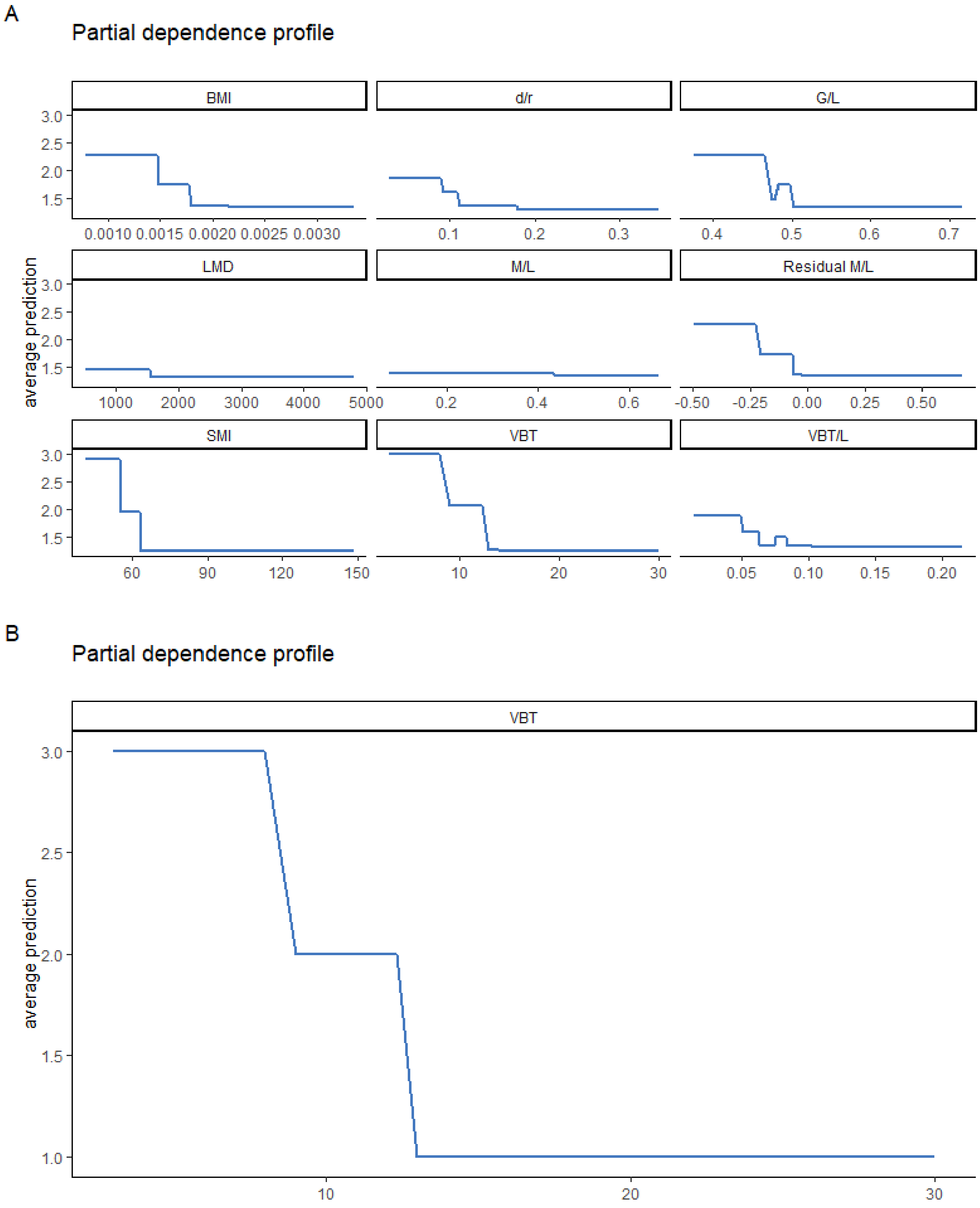

Figure S2: Univariate partial dependence plots of A) all variables and their prediction using the optimal model and B) using the reduced model.

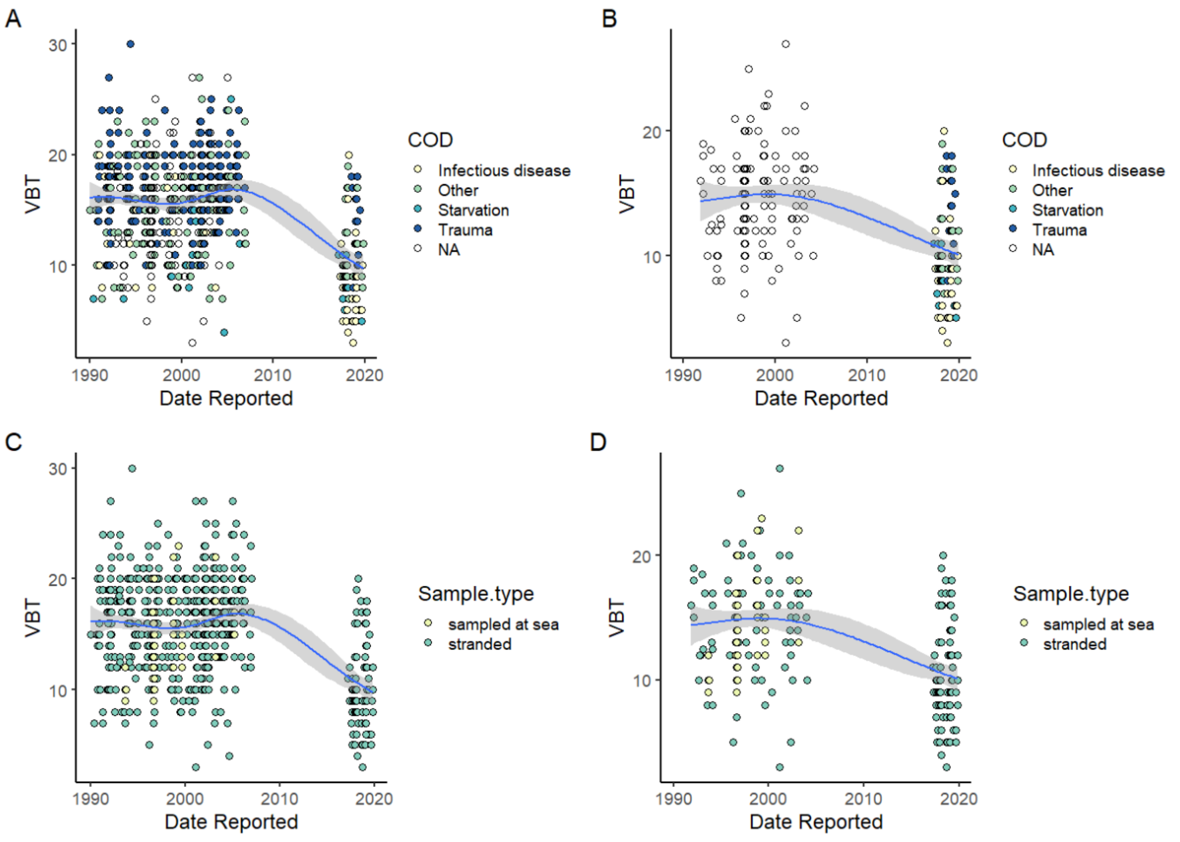

Figure S3: Ventral blubber thickness (mm) from 1990 to 2019 per A and C) All data, B and D) Irish historical and contemporary, only. Sample points were colour coded after A and B) causes of death and C and D) A general additive model smoothed curve is displayed with 95% confidence intervals.

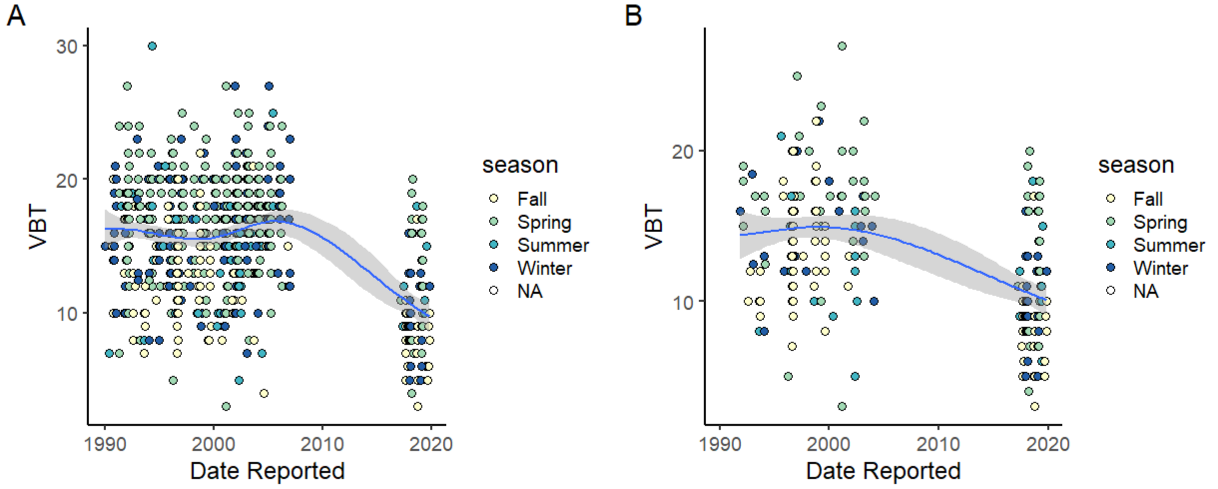

Figure S4: Ventral blubber thickness (mm) from 1990 to 2019 per A) All data, B) Irish historical and contemporary, only. Sample points were colour coded after seasons. A general additive model smoothed curve is displayed with 95% confidence intervals.

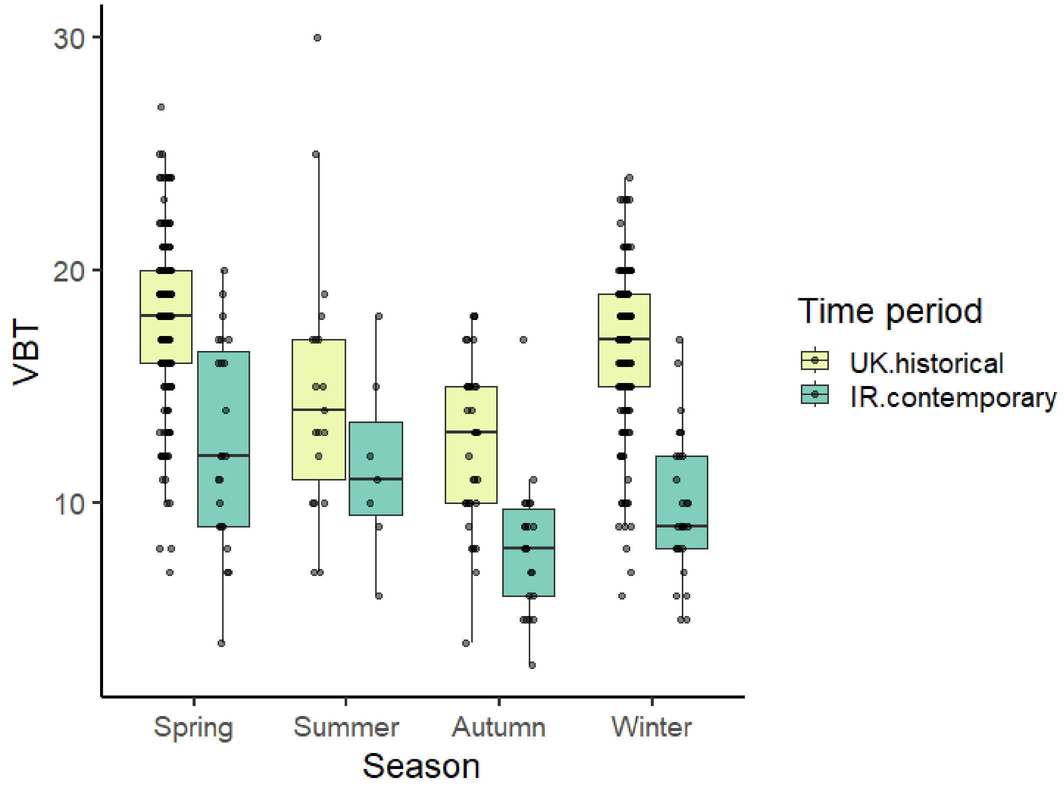

Figure S5: VBT (mm) across seasons per data set.

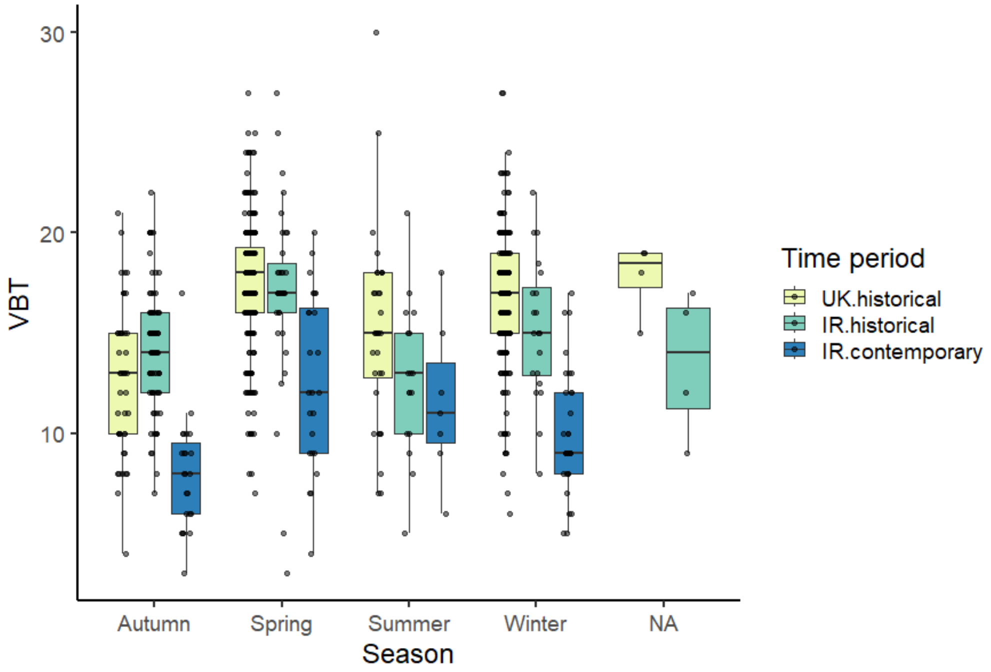

Figure S6: VBT (mm) across seasons per data set.

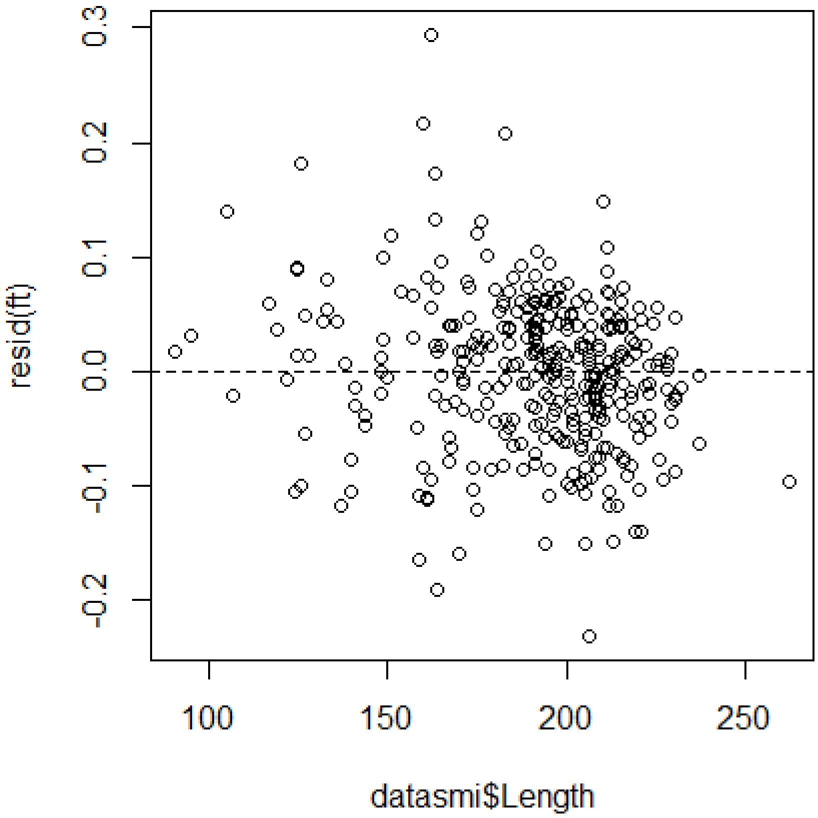

Figure S7: Residuals of the SMA regression of mass against body length (cm) populated during the calculation of the SMI.
